# SMAD4 feedback regulates the canonical TGF-β signaling pathway to control granulosa cell apoptosis

**DOI:** 10.1038/s41419-017-0205-2

**Published:** 2018-02-02

**Authors:** Xing Du, Zengxiang Pan, Qiqi Li, Honglin Liu, Qifa Li

**Affiliations:** College of Animal Science and Technology, Nanjing Agricultural University, Nanjing, 210095 China

## Abstract

Canonical TGF-β signals are transduced from the cell surface to the cytoplasm, and then translocated into the nucleus, a process that involves ligands (TGF-β1), receptors (TGFBR2/1), receptor-activated SMADs (SMAD2/3), and the common SMAD (SMAD4). Here we provide evidence that SMAD4, a core component of the canonical TGF-β signaling pathway, regulates the canonical TGF-β signaling pathway in porcine granulosa cells (GCs) through a feedback mechanism. Genome-wide analysis and qRT-PCR revealed that SMAD4 affected miRNA biogenesis in GCs. Interestingly, TGFBR2, the type II receptor of the canonical TGF-β signaling pathway, was downregulated in SMAD4-silenced GCs and found to be a common target of SMAD4-inhibited miRNAs. miR-425, the most significantly elevated miRNA in SMAD4-silenced GCs, mediated the SMAD4 feedback regulation of the TGF-β signaling pathway. This was accomplished through a direct interaction between the transcription factor SMAD4 and the miR-425 promoter, and a direct interaction between miR-425 and the TGFBR2 3′-UTR. Furthermore, miR-425 enhanced GC apoptosis by targeting TGFBR2 and the canonical TGF-β signaling pathway, which was rescued by SMAD4 and TGF-β1. Overall, our findings demonstrate that a positive feedback mechanism exists within the canonical TGF-β signaling pathway. This study also provides new insights into mechanism underlying the canonical TGF-β signaling pathway, which regulates GC function and follicular development.

The transforming growth factor (TGF)-β signaling pathway is one of the most important signaling pathways. It is not only expressed in all organisms but it is also involved in the regulation of most cellular and molecular processes during development and disease^1,2^. Nevertheless, the canonical TGF-β signaling pathway is relatively simple^3^. Briefly, TGF-β ligands (e.g., TGF-β1) first interacts with TGFBR2 (type II receptor) on the cell surface, and then phosphorylates TGFBR1 (type I receptor), signal is transduced from the cell surface to the cytoplasm. In the cytoplasm, phosphorylated TGFBR1 activates SMAD2/3 intracellular signaling, thus forming heteromeric complexes with SMAD4, and translocated into the nucleus. In the nucleus, the SMAD complex interacts with other transcription factors such as FOXL2^4^, or co-activators such as SMIF^5^, p300/CBP^6^, and BRD7^7^, or co-repressors such as TGIF^8^ and SnoN^9^ to regulate the transcription of targets by binding to target promoters termed SMAD-binding elements (SBEs), thus promoting signal transmission.

In organisms, the TGF-β signaling pathway is controlled by multiple factors such as microenvironmental condition^10,11^, hormones^12^, cytokines and growth factors^13^, microRNAs (miRNAs)^14^, long non-coding RNAs^15^, kinases for phosphorylation and dephosphorylation^16^, ubiquitin ligases and de-ubiquitinating enzymes^9,17^, and other factors^3^. Notably, the feedback regulation of the TGF-β signaling pathway involves downstream molecules such as SMAD7, one of the inhibitory Smads (I-Smads) and a negative regulator of the TGF-β signaling pathway^18^. SMAD7 is not a component of the canonical TGF-β signaling pathway, but it is the most studied feedback regulator of the canonical TGF-β signaling pathway (for review, see ref. ^18^). SMAD7 antagonizes the canonical TGF-β signaling pathway in many ways, such as by interfering with interactions between SMAD2/3 and TGFBR1, degrading TGFBR1 in cooperation with other regulators (e.g., SMURF2), and preventing complex formation between SMAD2/3 and SMAD4. Among the SMAD proteins (SMAD2/3/4) of the canonical TGF-β signaling pathway, although observed that endogenous TGF-β1 expression was induced in SMAD2 transgenic mice^19^, and TGF-β1 mRNA level was reduced in Smad3-null mice^20^, the mechanism by these SMAD proteins feedback regulation of TGF-β1 is not known. In this study, we sough to understand the feedback mechanism in the canonical TGF-β signaling pathway.

## Results

### Genome-wide analysis reveals SMAD4 regulation of miRNA biogenesis in GCs

**Fig. 1:**
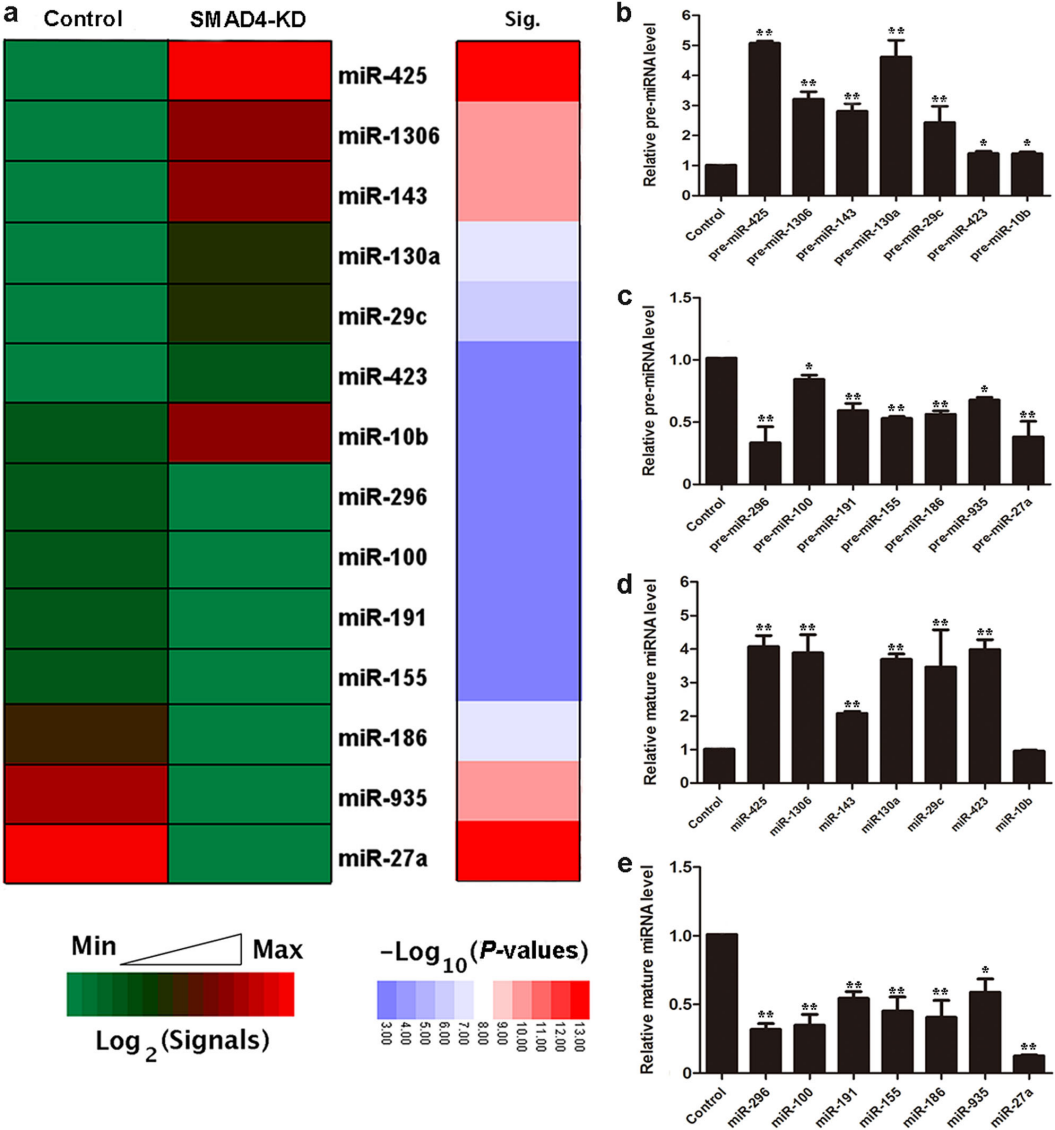
Differentially expressed (DE) miRNAs in response to SMAD4 silencing in porcine GCs. **a** Heat map of SMAD4-dependent miRNAs. Heat map showing the signals of DE miRNAs in SMAD4-silenced GCs based on previously published RNA-Seq data^21^. KD, knockdown. **b**, **c** SMAD4-siRNA influences pre-miRNA expression in GCs. GCs were transfected with SMAD4-siRNA, the levels of SMAD4-inhibited pre-miRNAs (**b**) and SMAD4-induced pre-miRNAs (**c**) were quantified by qRT-PCR. **d**, **e** SMAD4-siRNA influences mature miRNA expression in GCs. GCs were transfected with SMAD4-siRNA, mature miRNA expression levels reduced by SMAD4 (**d**) and induced by SMAD4 (**e**) were detected by stem-loop reverse-transcribed qRT-PCR. U6 served as the endogenous control. Experiments were conducted in triplicate. Error bars ± S.E.M. *P < 0.05; **P < 0.01

The present study was initiated in an attempt to identify SMAD4 targets in porcine GCs, and identified 1025 differentially expressed mRNAs (Supplementary Figure S1a) in SMAD4-siRNA-treated GCs by RNA-Seq^21^. Interestingly, we also found 14 differentially expressed pre-miRNAs, including seven upregulated pre-miRNAs and seven downregulated pre-miRNAs, in SMAD4-silenced GCs (Fig. 1a). After verifying these results quantitative real-time PCR (qRT-PCR; Fig. 1b, c) indicated that SMAD4 controls miRNA biogenesis in GCs.

As a negative regulator, miRNA silences targets at the post-transcriptional level (inhibits mRNA translation or degrades mRNAs) by directly interacting with its mature sequence (mainly the seed sequence) and targeting mRNA 3′-untranslated region (UTR)^1,22^. To test whether SMAD4 affects these differentially expressed miRNAs, we quantified the mature miRNA expression levels in SMAD4-silenced GCs. Seven downregulated pre-miRNAs had similar expression patterns as mature miRNAs in control and SMAD4-siRNA-treated GCs (Fig. 1d). Among the seven upregulated pre-miRNAs, six pre-miRNAs had similar expression patterns as mature miRNAs, except that mature miR-10b did not show a significant difference (Fig. 1e). These results indicate that SMAD4 is involved in the function of SMAD4-dependent miRNAs (differentially expressed miRNAs) in GCs.

### TGFBR2 is a common target of SMAD4-inhibited miRNAs

**Fig. 2:**
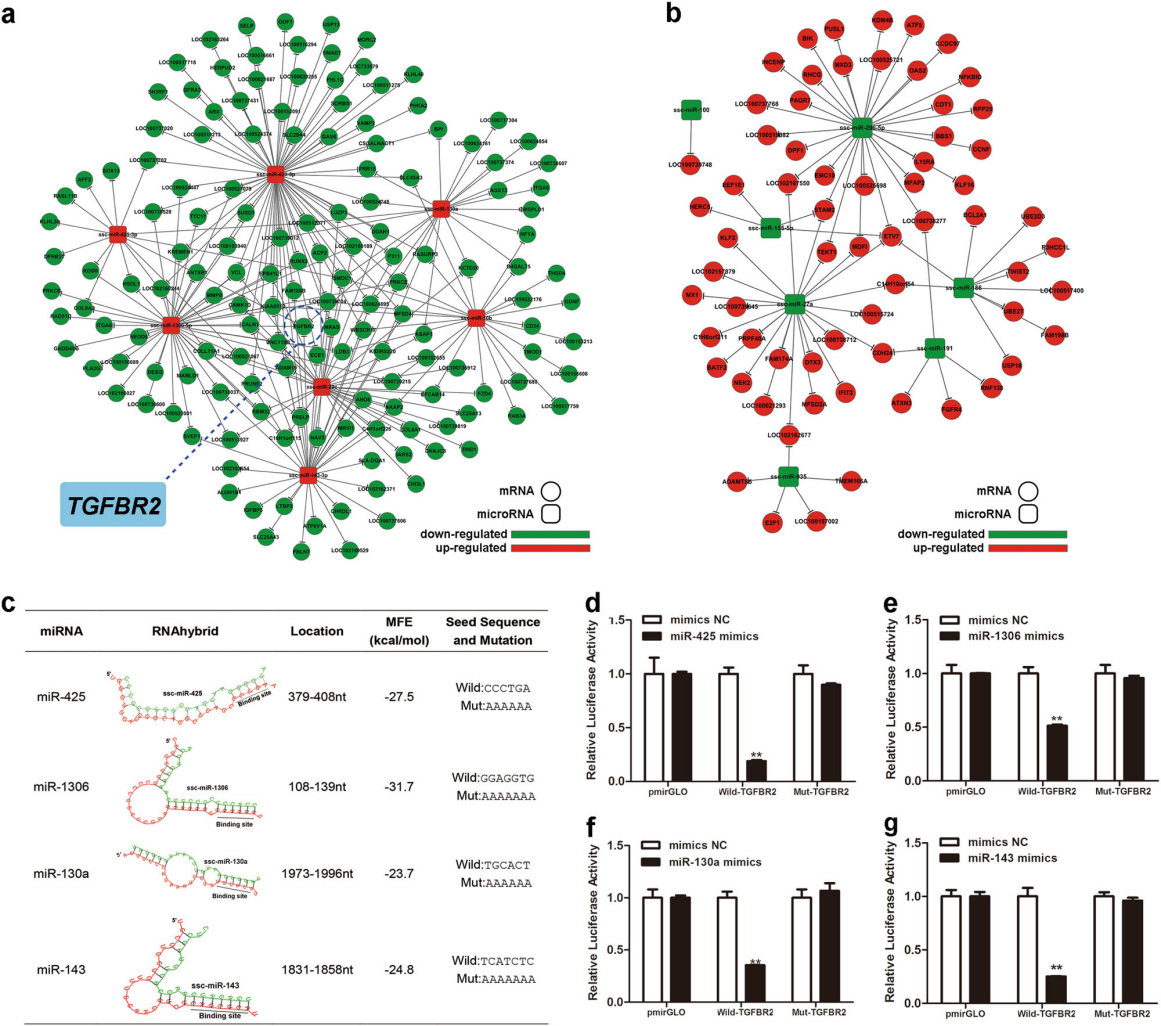
*TGFBR2* is a common target of SMAD4-inhibited miRNAs. **a** miRNA-mRNA interaction network for SMAD4-inhibited miRNAs. **b** miRNA-mRNA interaction network for SMAD4-inhibited miRNAs. **c** The miRNA response element (MRE) of four SMAD4-inhibited miRNAs was identified in the porcine *TGFBR2* 3′-UTR. **d**–**g** The porcine *TGFBR2* gene is a direct target of SMAD4-inhibited miRNAs. HEK 293 cells were co-transfected with miRNA mimics and a construct carrying the *TGFBR2* 3′-UTR with a wild-type or mutated MRE motif, and luciferase activity was measured. Average results from three independent experiments are shown. Error bars ± S.E.M. **P < 0.01

To understand the function and regulatory mechanisms of the differentially expressed miRNAs, a SMAD4-dependent miRNA-mRNA network was generated. The miRNA-mRNA interaction network revealed that 147 mRNAs were candidate targets of the SMAD4-inhibited miRNAs (Fig. 2a, Supplementary Figure S1b, and Supplementary Table S1), whereas 65 mRNAs were candidate targets of the SMAD4-induced miRNAs (Fig. 2b, Supplementary Figure S1c, and Supplementary Table S2). Notably, of 7 SMAD4-inhibited miRNAs, 4 miRNAs (miR-425, miR-1306, miR-130a, and miR-143) targeted TGFBR2 (LOC100038019), upstream of SMAD4 in the canonical TGF-β signaling pathway, and a transcript that is downregulated in SMAD4-silenced GCs^21^.

The putative miRNA response elements (MREs) of these four miRNAs (miR-425, miR-1306, miR-130a, and miR-143) were all detected within the 3′-UTR of the porcine *TGFBR2* gene (positions 379–408, 108–139, 1973–1996, and 1831–1858, respectively; Fig. 2c). Minimum free energy (MFE) analysis also revealed that all of the four miRNAs had a high binding capacity with the 3′-UTR of porcine *TGFBR2* gene by RNAhybrid (Fig. 2c). In addition, the mature and seed sequences of these four miRNAs are evolutionary conserved in vertebrates, including humans (Supplementary Figures S2).

To test whether *TGFBR2* is a target of these four miRNAs, we constructed a dual-luciferase reporter vector containing the wild-type MRE located in the 3′-UTR of TGFBR2 or a mutated version of the MRE, respectively (Fig. 2c and Supplementary Figures S3), followed by co-transfection with miRNA mimics into porcine GCs. Luciferase activity assay results showed that four miRNAs (miR-425, miR-1306, miR-130a, and miR-143) all significantly decreased wild-type reporter activity, whereas they could not affect the activity of the mutant reporter (Fig. 2d–g), indicating that *TGFBR2* is a common and direct target of these four SMAD4-inhibited miRNAs.

### miR-425 enhances GC apoptosis through the canonical TGF-β signaling pathway by targeting TGFBR2

**Fig. 3:**
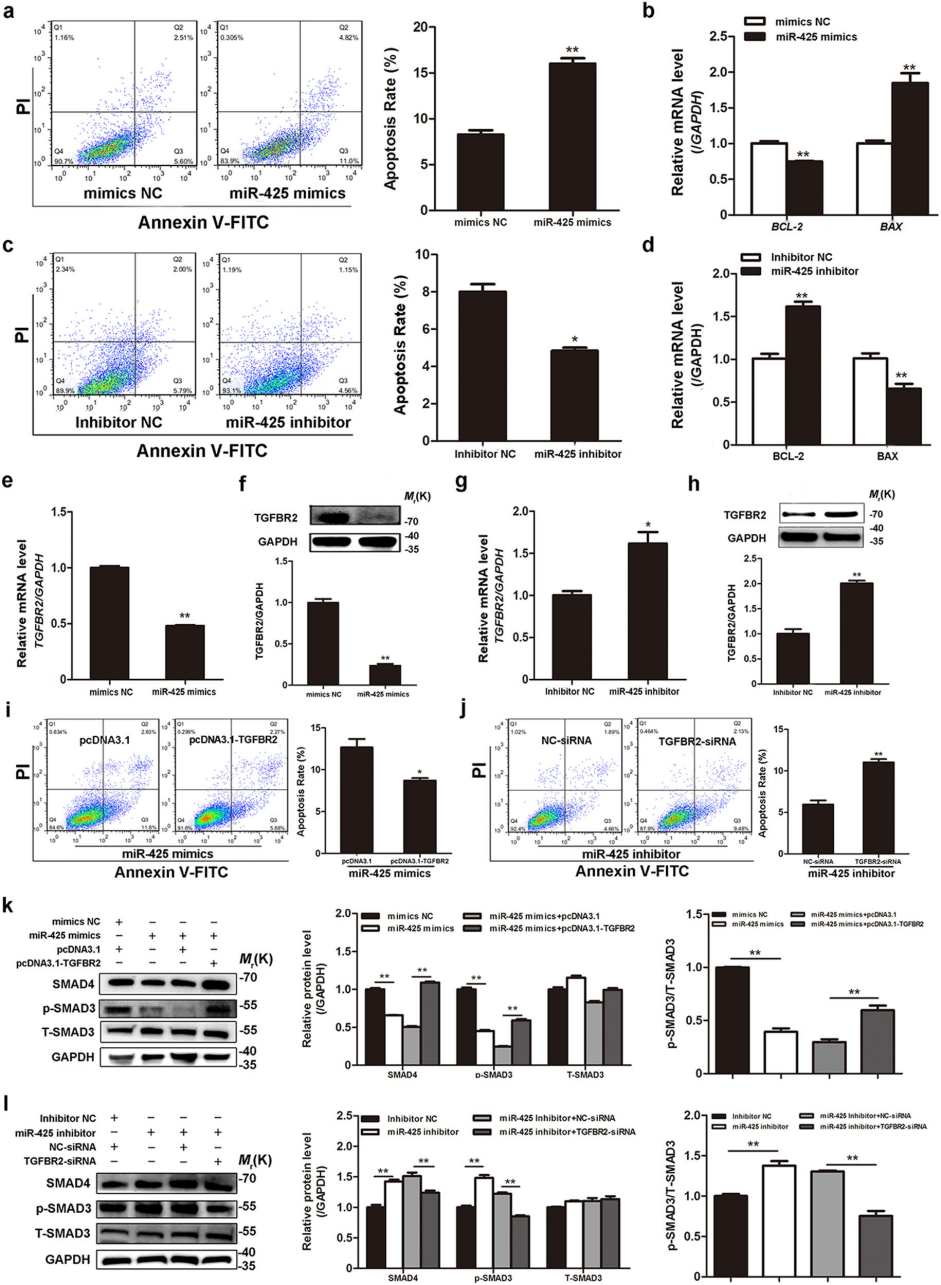
miR-425 is an epigenetic factor of porcine GC apoptosis, and functions by targeting TGFBR2. **a**, **b** miR-425 enhances GC apoptosis. GCs were treated with miR-425 mimics, cell apoptosis was detected by FACS (**a**, left), the apoptosis rate was calculated (**a**, right), and the mRNA levels of *BCL-2* and *BAX* were detected by qRT-PCR (**b**). **c**, **d** Silencing of miR-425 inhibits GC apoptosis. GCs were treated with miR-425 inhibitor, cell apoptosis was detected by FACS (**c**), and the mRNA levels of *BCL-2* and *BAX* were detected by qRT-PCR (**d**). **e**–**h** miR-425 controls endogenous TGFBR2 expression in GCs. GCs were transfected with miR-425 mimics or inhibitor, TGFBR2 mRNA levels were measured by qRT-PCR (**e**, **g**), and TGFBR2 protein levels were quantified by western blotting (**f**, **h**). **i**–**l** miR-425 controls GC apoptosis and the canonical TGF-β signaling pathway by inhibiting TGFBR2. GCs were co-transfected with miR-425 mimics and pcDNA3.1-TGFBR2, or miR-425 inhibitor and TGFBR2-siRNA, cell apoptosis was calculated by FACS (**i**, **j**), and protein levels of members within the canonical TGF-β signaling pathway were detected by western blotting (**k**, **l**). The results represent the average of three independent experiments. Error bars ± S.E.M. *P < 0.05; **P < 0.01

As the most significantly elevated miRNA in SMAD4-silenced GCs, miR-425 was chosen for further analysis. However, its role in ovary remains unclear. A fluorescence-activated cell sorting (FACS) assay showed that overexpression of miR-425 increased the apoptosis rate (Fig. 3a) and BAX expression, and decreased BCL-2 expression, in porcine GCs (Fig. 3b), whereas inhibition of miR-425 decreased the apoptosis rate (Fig. 3c) and BAX expression, and increased BCL-2 expression (Fig. 3d). Based on these findings, we concluded that miR-425 is a pro-apoptotic factor in GCs, which is essential for GC function.

We next explored the mechanism underlying the miR-425-mediated control of GC apoptosis. Ectopic expression of miR-425 in porcine GCs downregulated TGFBR2 mRNA and protein levels (Fig. 3e, f), whereas inhibition of miR-425 upregulate TGFBR2 mRNA and protein levels (Fig. 3g, h). Moreover, TGFBR2 rescued miR-425-induced GC apoptosis and suppressed miR-425 inhibitor-caused downregulation of the apoptosis rate (Fig. 3i, j). Taking these results together, we demonstrated that miR-425 controls GC apoptosis by target inhibiting endogenous TGFBR2.

We further elucidated whether miR-425 targets TGFBR2 to regulate the TGF-β signaling pathway in porcine GCs. miR-425 overexpression resulted in decreases in the protein levels of phospho-SMAD3 (p-SMAD3), the active marker and downstream member of the TGF-β signaling pathway, and SMAD4 (Fig. 3k), similarly to TGFBR2 in miR-425-overexpressing GCs. Accordingly, an inhibition of miR-425 increased p-SMAD3 and SMAD4 protein levels in GCs (Fig. 3l). We also investigated whether TGFBR2 mediated miR-425 regulation of the TGF-β signaling pathway in GCs. TGFBR2 overexpression rescued the miR-425-mediated decrease in p-SMAD3 and SMAD4 protein levels, whereas TGFBR2 knockdown suppressed the miR-425 inhibitor-mediated increase in p-SMAD3 and SMAD4 protein levels in GCs (Fig. 3k, l), demonstrating that miR-425 regulates the TGF-β signaling pathway through TGFBR2.

### SMAD4 regulates miR-425 expression by directly binding to its promoter

**Fig. 4:**
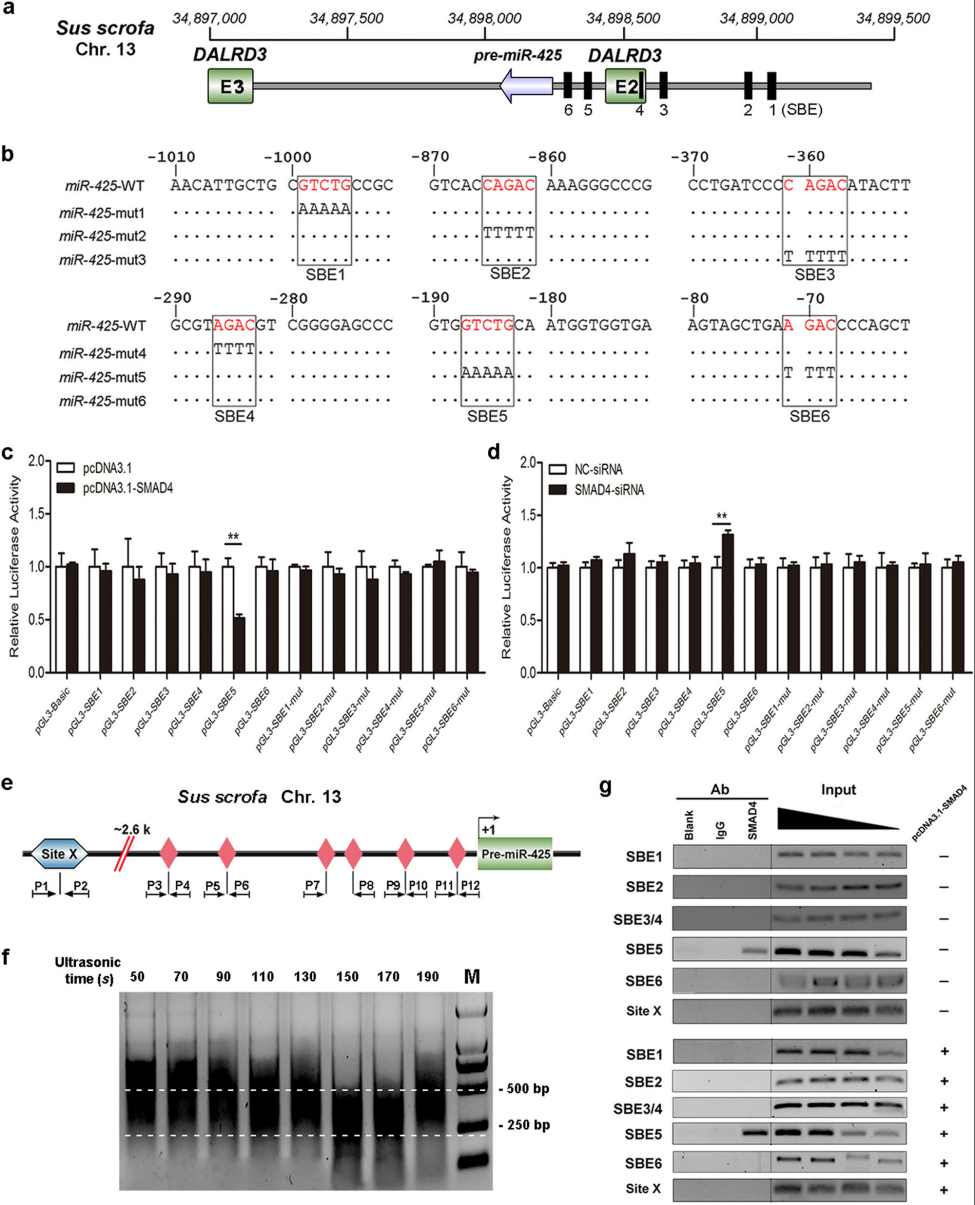
SMAD4 decreases miR-425 expression in porcine GCs by directly binding to its promoter. **a** Schematic diagram showing the genome location of the miR-425 and potential SMAD-binding sites (SBEs, black boxes). **b** The luciferase reporter vector of the porcine miR-425 promoter. **c**, **d** SMAD4 regulates miR-425 promoter activity. GCs were co-transfected with pcDNA3.1-SMAD4 or SMAD4-siRNA, and a construct carrying wild-type or mutated SBE motifs, and luciferase activity was determined. **e** Schematic diagram showing the miR-425 promoter region. P1–P12: primers used for the ChIP assay. Site X, which represents the negative control locus for the ChIP assay. **f** Crosslinked chromatins from GCs were sonicated from 50 to 190 s to acquire the appropriate chromatin size and identify the optimal ultrasonic time for the ChIP assay. **g** ChIP assays. Input titrations are shown for each chromatin preparation (50, 25, 12.5, and 6.25%). Experiments were conducted in triplicate. Error bars ± S.E.M. **P < 0.01

To examine the SMAD4-mediated mechanism of miR-425 inhibition in porcine GCs, the promoter of porcine miR-425 was identified and characterized (Supplementary Figures S4). Six SBE motifs were detected in the promoter region of porcine miR-425 (Fig. 4a and Supplementary Figures S5). Luciferase assay showed that a stimulation of SMAD4 decreased the activity of the miR-425 promoter containing the SBE5 motif (Fig. 4b, c), whereas an inhibition of SMAD4 increased the activity of the miR-425 promoter containing the SBE5 motif (Fig. 4d). By contrast, there was no change in the activity of the SBE5-mutated promoter (Fig. 4c, d). However, both the overexpression and knockdown of SMAD4 could not affect the activity of the miR-425 promoter containing other wide-type or mutated SBE motifs (Fig. 4c, d). Furthermore, chromatin immunoprecipitation (ChIP) assay demonstrated that SMAD4 could directly bind to the SBE2 motif of the miR-425 promoter, but not to other SBE motifs (Fig. 4e–g). Together, these data provide compelling evidence demonstrating SMAD4 inhibits the transcriptional activity of the *miR-425* gene in porcine GCs by directly binding to the SBE5 motif of the miR-425 promoter.

### miR-425 mediated SMAD4 feedback regulation of the canonical TGF-β signaling pathway

**Fig. 5:**
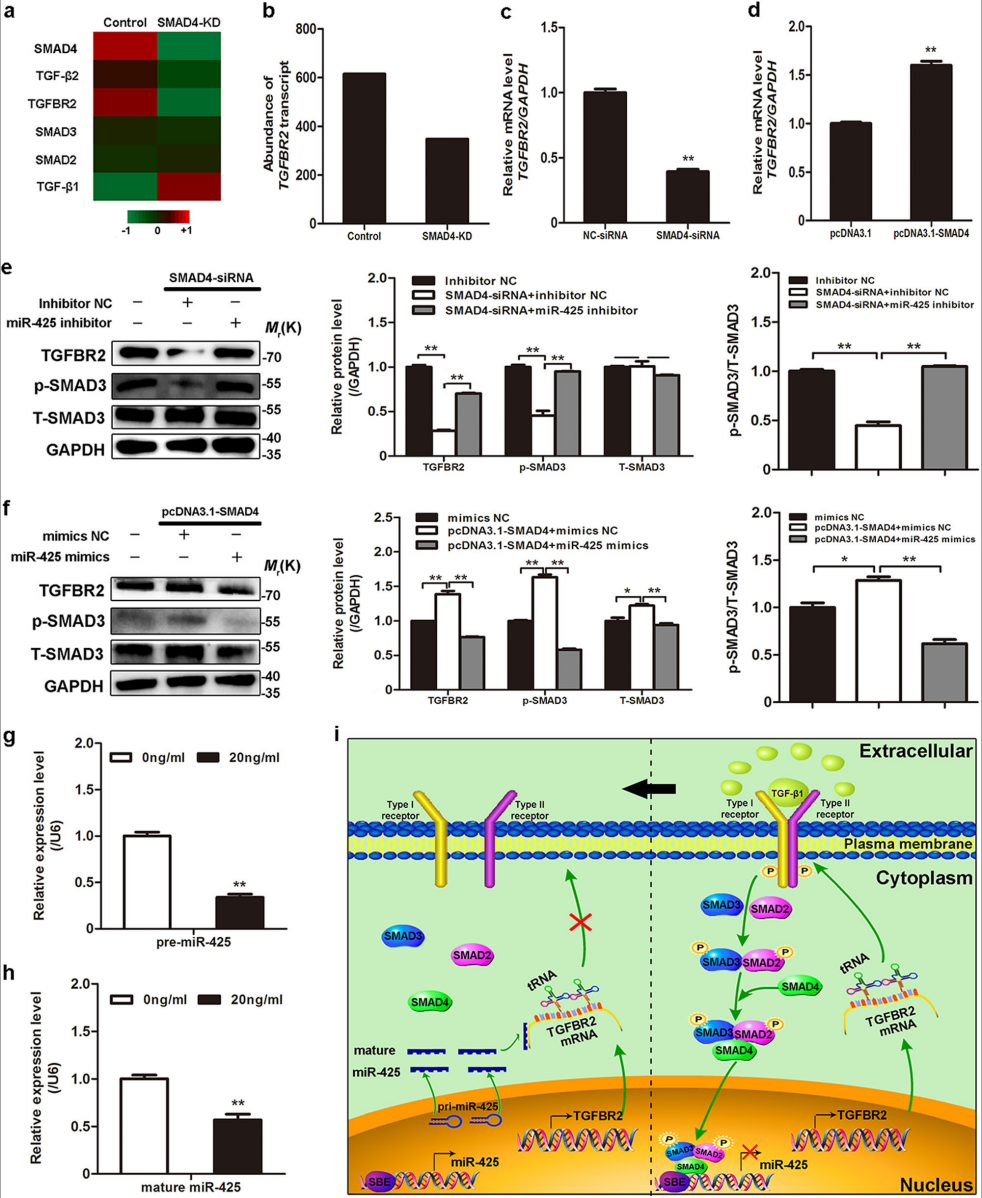
miR-425-mediated SMAD4 feedback regulation of the canonical TGF-β signaling pathway. **a** Heat map for DE molecules of the canonical TGF-β signaling pathway in porcine GCs treated with SMAD4-siRNA (SMAD4-KD) or NC siRNA (control). Data were obtained from RNA-Seq data^21^. **b** Abundance of *TGFBR2* transcript in control and SMAD4-KD groups using RNA-Seq data. **c** GCs were transfected with SMAD4-siRNA, and TGFBR2 mRNA levels were detected by qRT-PCR. **d** GCs were transfected with pcDNA3.1-SMAD4, and detected by qRT-PCR. **e** GCs were co-treated with SMAD4-siRNA and miR-425 inhibitor, and the protein levels were analyzed by western blotting. **f** GCs were co-treated pcDNA3.1-SMAD4 and miR-425 mimics, and analyzed by western blotting. **g** GCs were treated with TGF-β1 (20 ng/ml), and pre-miR-425 levels were quantified by qRT-PCR. **h** GCs were treated with TGF-β1, and mature miR-425 levels were quantified by stem-loop reverse-transcribed qRT-PCR. **i** A model for miR-425-mediated SMAD4 feedback regulation of the canonical TGF-β signaling pathway in porcine GCs. The results represent the average of three independent experiments. Error bars ± S.E.M. *P < 0.05; **P < 0.01; ns, no significant difference

Results from an earlier, as well as the present, study showed that TGFBR2, an upstream molecule of SMAD4, was downregulated in SMAD4-silenced GCs by previously published RNA-Seq data (Fig. 5a, b)^21^. qRT-PCR confirmed that SMAD4 knockdown decreased TGFBR2 mRNA expression in GCs (Fig. 5c), whereas SMAD4 overexpression increased its expression (Fig. 5d). Similarly, western blotting showed that SMAD4 also increased TGFBR2 protein expression in GCs (Fig. 5e, f), indicating that SMAD4 positively regulates TGFBR2 expression in GCs. As discussed above, our results demonstrate for the first time that SMAD4, a core component of the canonical TGF-β signaling pathway, regulates this pathway through a feedback mechanism.

Next, we investigated the SMAD4 feedback mechanism regulating the canonical TGF-β signaling pathway. miR-425 is a functional target of SMAD4 and a direct inhibitor of TGFBR2 in GCs, leading us to speculate that miR-425 might mediate the SMAD4 feedback regulation of the canonical TGF-β signaling pathway. To confirm this, the SMAD4-specific siRNA and miR-425 inhibitor were co-transfected into porcine GCs. The results showed that the miR-425 inhibitor rescued the SMAD4-specific siRNA-mediated decrease in TGFBR2 and p-SMAD3 protein expression (Fig. 5e). By contrast, miR-425 overexpression inhibited the SMAD4-mediated increase in TGFBR2 and p-SMAD3 protein expression (Fig. 5f), indicating that miR-425 promotes the SMAD4 feedback regulation of the canonical TGF-β signaling pathway.

## Discussion

SMAD4 is a core component of the canonical TGF-β signaling pathway, and TGF-β signals are ultimately transmitted into the nucleus with the help of SMAD4. Thus, an in-depth analysis of SMAD4 targets is a key channel for understanding the molecular mechanism by the canonical TGF-β signaling pathway controls cell function^21,23–25^. For example, 2518, 1025, and 941 targets were identified in mouse embryonic stem cells by employing ChIP-chip technology^24^, in porcine GCs by sequencing technology (RNA-Seq)^21^, and in the stomach of Smad4^+/−^ mutant mice by Sleeping Beauty transposon mutagenesis^25^, respectively. SMAD4 and SMAD4-dependent canonical TGF-β signaling pathway is essential for the fate (growth or atresia) of follicles, ovulation, and female reproduction in mammals^26,27^. In ovarian GCs, we previously performed a genome-wide screening for targets of SMAD4 in porcine GCs^21^. In the present study, we further showed, at the genome-wide level, that SMAD4 controls GC and ovarian function through influencing miRNA biogenesis. Consistent with this, Davis et al.^1,28^ demonstrated that SMAD proteins control DROSHA-mediated miRNA maturation in human smooth muscle cells. Meanwhile, the regulation of individual miRNA by SMAD4 has been reported In GCs^22^. Our findings expands these results and demonstrated that SMAD4 is involved in miRNA biogenesis, such as transcription and maturation, in GCs at the genome-wide level. In addition, we also showed that the SMAD4-dependent miRNAs such as miR-425 also regulated by the canonical TGF-β signaling pathway (Fig. 5g, h). Several miRNAs that respond to TGF-β signals have recently been identified in GCs of mammals such as mice^29^ and pigs^22^. Our findings also provides a new mechanistic approach to study the canonical TGF-β signaling pathway in GCs of mammals, including humans. However, additional studies are needed to understand the functions of non-coding regulatory RNAs, including miRNAs, lncRNAs, and circular RNAs, that interact with the canonical TGF-β signaling pathway.

Our mechanism studies showed that TGFBR2 is a common target of SMAD4-dependent miRNAs, including miR-425. TGFBR2 is the first receptor to be activated within the TGF-β signaling pathway, and plays an important role in TGF-β signal transduction. A recent report also demonstrated that miR-425 directly binds to the 3′-UTR of the human *TGFBR2* gene^30^. Furthermore, dozens of miRNAs that known to regulate TGFBR2 expression, such as miR-21^31,32^, miR-145^33^, miR-211^34^, miR-130a-3p^35^, and miR-17∼92 family clusters^36^, in several animal species, including humans, rodents, domestic animals, and fish. This study is the first report on the identification of miRNAs that target TGFBR2 in pigs. In addition, in the human, miR-425 targets not only TGFBR2 but also SMAD2, a component of the canonical TGF-β signaling pathway and a downstream molecule of TGFBR2^30,37^. However, our study did not show that *SMAD2* is the target gene for miR-425 in pigs (Supplementary Figures S6), suggesting that miR-425 controls the canonical TGF-β signaling pathway by targeting different component in humans (e.g., TGFBR2 and SMAD2) and pigs (e.g., TGFBR2).

miR-425 is an intronic RNA, which is involved in multiple cellular physiological process, such as cell apoptosis, proliferation, migration, and invasion^38,39^, but mostly limited in cancer cells. In addition, miR-425 was identified as a potential diagnostic biomarker for cancer and other diseases such as Alzheimer’s disease^40,41^. There is a correlation between TGFBR2 and ovarian cancer risk^42^, but it is unclear whether miR-425 can serve as a novel biomarker for diagnosis of early-stage ovarian cancer. Here we demonstrated that miR-425 controls GC apoptosis by directly inhibiting endogenous TGFBR2. This in stark contrast to role of its regulator SMAD4 in porcine GCs^22,27^ and function of its target TGFBR2 in mouse follicular development^43^. Furthermore, several studies reported that miRNAs regulate follicle development through TGFBR2. For example, Yang et al.^43^ showed that miR-145 targets Tgfbr2 to initiate of primordial follicle development and maintain primordial follicle quiescence in the neonatal mouse ovary.

Importantly, we demonstrate for the first time that SMAD4, a center component of the canonical TGF-β signaling pathway, regulates this pathway through a feedback mechanism. Previous studies illustrated that SMAD2 and SMAD3, other proteins of the canonical TGF-β signaling pathway, also regulates TGF-β1, the ligand of the canonical TGF-β signaling pathway in cells^19,20^. This study not only fills an important gap within regulation of the canonical TGF-β signaling pathway but also consummated to the SMAD proteins (SMAD2, SMAD3, and SMAD4) belong to the canonical TGF-β signaling pathway, are all positive feedback regulation their upstream signaling molecule, together with the former proved SMAD2 and SMAD3 feedback enhance TGF-β1^19,20^. Furthermore, SMAD7 also inhibits the canonical TGF-β signaling pathway through a feedback mechanism^18^, indicating that all the canonical TGF-β signaling pathway-related SMAD proteins (SMAD2, SMAD3, SMAD4, and SMAD7) maybe share a feedback regulatory function. However, the mechanism by SMAD2/3 induction of their ligand TGF-β1 are needed to further research.

In summary, we provide evidence that the existence of a feedback mechanism within the canonical TGF-β signaling pathway in ovarian GCs of mammals (Fig. 5i). SMAD4, a core component of the canonical TGF-β signaling pathway, was identified for the first time as a novel feedback regulator. Moreover, our finding also demonstrated miRNAs interact with the canonical TGF-β signaling pathway to control GC apoptosis, provides new insights into mechanism underlying the canonical TGF-β signaling pathway regulates GC function and follicular development.

## Materials and Methods

### Cell culture and transfection

Fresh porcine ovaries were obtained and transported to the laboratory within 1 h. GCs were collected and cultured as previously described^22^. HEK 293 cells were maintained at 37 °C and either 5% CO_2_ in Dulbecco’s modified Eagle medium (Sigma) with 10% fetal bovine serum. For transfection, cells were seeded into 6-well or 12-well plates for 12 h and relative plasmids or oligonucleotides were transfected using Lipofectamine 2000 (Invitrogen) according to the manufacturer’s protocol. The oligonucleotide sequences of miRNA mimics, inhibitors, and small-interference RNAs used are shown in Supplementary Table S3. Animal experiments were approved by the Animal Ethics Committee at Nanjing Agricultural University, China.

### RNA isolation and qRT-PCR

For miRNA detection, total RNA was isolated from porcine GCs using a High Purity Total RNA Extraction Kit (Qiagen), and reverse-transcribed into complementary DNA (cDNA) by using PrimeScript^®^ miRNA qPCR Starter Kit (TaKaRa). qRT-PCR was performed by using SYBR Green Master Mix (Vazyme Biotech Co., Ltd) on the StepOne Plus System (Applied Biosystems). The expression levels of pre- and mature miRNAs were then measured, and normalized to U6 small nuclear RNA. For coding gene detection, total RNA was reverse-transcribed into cDNA using PrimeScript^TM^ RT Master Mix (TaKaRa). qRT-PCR was performed in triplicate and normalized to *GAPDH*. The primers sequences for qRT-PCR are listed in Supplementary Table S4.

### Plasmids

The pcDNA3.1-SMAD4 plasmid was generated previously by our group^28^. For 3′-UTR luciferase reporters, the 3′-UTR fragments of TGFBR2 containing putative target sites of miRNAs (miR-425, miR-1306, miR-130a, and miR-143) were amplified from porcine genomic DNA and verified by sequencing. After digestion with *Xho*I and *Hind*III, fragment was cloned into pmirGLO Dual-luciferase miRNA Target Expression Vector (Promega). For 5′-UTR luciferase reporters, miR-425 promoter was amplified and cloned into pGL-3 reporter vector (Promega) within *Kpn*I and *Xho*I sites. Site-directed mutagenesis kit (TaKaRa, Dalian, China) was used to generate the mutant plasmids according to the manufacturer’s instructions. All mutants were verified by sequencing. Primers used here are detailed in Supplementary Table S5.

### Bioinformatic analysis

Differential expressed genes, including coding genes and miRNAs in SMAD4-silenced GCs were identified using RNA-Seq as described in our previous study^21^. The targets of miRNAs were predicted by three algorithms miRDB (http://www.mirdb.org/miRDB/), microRNA.org (http://www.microrna.org/), and miRWalk 2.0 database (http://zmf.umm.uni-heidelberg.de/apps/zmf/mirwalk2/). The SMAD4-dependent miRNA-mRNA interaction networks were generated by cytoscape software (download from http://cytoscape.org). RNAhybrid (http://bibiserv.techfak. uni-bielefeld.de/rnahybrid/) was performed to predict the putative MRE in the 3′-UTR of TGFBR2, and the MFE. Pre- and mature sequences of miRNAs were obtained from miRBase (http://www.mirbase.org/).

### Luciferase reporter assays

Cells were harvested and the lysates were collected 24 h post transfection, firefly and *Renilla* luciferase activities were measured by using a Dual-Luciferase Reporter Assay System (Promega) according to the manufacturer’s instruction. Relative luciferase activity indicates the ratio of firefly luciferase activity to *Renilla* luciferase control.

### Western blots

Antibodies used for western blotting include TGFBR2 (Santa Cruz #sc-400, 1:1000), SMAD3 (Santa Cruz #sc-8332, 1:1000), SMAD4 (Santa Cruz #sc-1909-R, 1:1000), phosphor-SMAD3 (Cell Signaling Technology #9520, 1:2000), and GAPDH (ORIGENE #TA802519, 1:5000). The method of western blot was described in detail in ref. ^22^.

### Apoptosis analysis

To measure the apoptosis rate of GCs, Annexin V-fluorescein isothiocyanate and propidium iodide were used according to the manufacturer’s instruction (Vazyme Biotech co., ltd). In total, 2 × 10^5^ cells were sorted by FACS with a cell counting machine (Becton Dickinson), and cells were analyzed using FlowJo software (TreeStar). The apoptosis rate was calculated using the following equation: (number of cells in the right lower quadrant + number of cells in the right upper quadrant)/(total number of cells).

### Chromatin immunoprecipitation

ChIP assays were performed as previously described^22^. Briefly, GCs were crosslinked with 1% formaldehyde for 10 min at room temperature and quenched in glycine. Then, cells were sonicated and DNA was immunoprecipitated from lysates using rabbit anti-SMAD4 antibody (Santa Cruz #sc-1909-R, 1:1000). Following, the purified DNA was subjected to PCR to amplify the SBEs in the promoter of miR-425. PCR-amplified products were analyzed on a 3% agarose gel. A nonspecific antibody against IgG (Santa Cruz #sc-2358, 1:1000) served as the negative control and the chromatin before immunoprecipitation was used as the input control. The PCR primers are listed in Supplementary Table S6.

### TGF-β1 treatment

Porcine GCs were seeded into six-well plates with 5 × 10^5^ cells per well for 12 h and then serum-starved for 16–24 h before adding porcine TGF-β1 (R&D Systems) at the final concentration to 20 ng/ml. After 24 h, cells were harvested and the expression levels of pre- and mature miR-425 were determined.

### Statistical analysis

All data are presented as means ± S.E.M. Prism 5 software (GraphPad Software) was used to perform statistical analysis. Two-tailed Student’s *t*-test was used to evaluate the significance when two groups were compared. When three or more groups were compared, a one-way analysis of variance test was performed and Turkey’s test to determine significance between groups. *P*-value of <0.05 and 0.01 were considered as significant and extremely significant difference, respectively.

## Electronic supplementary material

Supplementary Figure

Supplementary Table

## References

[1] Davis BN, Hilyard AC, Lagna G, Hata A (2008). SMAD proteins control DROSHA-mediated microRNA maturation. Nature.

[2] Granata A (2017). An iPSC-derived vascular model of Marfan syndrome identifies key mediators of smooth muscle cell death. Nat. Genet..

[3] Hata A, Chen YG (2016). TGF-beta signaling from receptors to Smads. Cold Spring Harb. Perspect. Biol..

[4] Fortin J, Boehm U, Deng CX, Treier M, Bernard DJ (2014). Follicle-stimulating hormone synthesis and fertility depend on SMAD4 and FOXL2. FASEB J..

[5] Bai RY (2002). SMIF, a Smad4-interacting protein that functions as a co-activator in TGFbeta signalling. Nat. Cell Biol..

[6] Liu S (2016). SUMO modification reverses inhibitory effects of Smad nuclear Interacting protein-1 in TGF-beta responses. J. Biol. Chem..

[7] Liu T (2017). Tumor suppressor bromodomain-containing protein 7 cooperates with Smads to promote transforming growth factor-beta responses. Oncogene.

[8] Wotton D, Lo RS, Lee S, Massague J (1999). A Smad transcriptional corepressor. Cell.

[9] Bonni S (2001). TGF-beta induces assembly of a Smad2-Smurf2 ubiquitin ligase complex that targets SnoN for degradation. Nat. Cell Biol..

[10] Ma B (2016). Zyxin-Siah2-Lats2 axis mediates cooperation between Hippo and TGF-beta signalling pathways. Nat. Commun..

[11] Barouch DH (2016). Rapid inflammasome activation following mucosal SIV infection of rhesus monkeys. Cell.

[12] Alonso-Merino E (2016). Thyroid hormones inhibit TGF-beta signaling and attenuate fibrotic responses. Proc. Natl Acad. Sci. USA.

[13] Demagny H, Araki T, De Robertis EM (2014). The tumor suppressor Smad4/DPC4 is regulated by phosphorylations that integrate FGF, Wnt, and TGF-beta signaling. Cell Rep..

[14] Yin S (2016). Differential TGFbeta pathway targeting by miR-122 in humans and mice affects liver cancer metastasis. Nat. Commun..

[15] Zhao JJ (2016). Long non-coding RNA ANRIL promotes the invasion and metastasis of thyroid cancer cells through TGF-beta/Smad signaling pathway. Oncotarget.

[16] Lin X (2006). PPM1A functions as a Smad phosphatase to terminate TGFbeta signaling. Cell.

[17] Dupont S (2009). FAM/USP9x, a deubiquitinating enzyme essential for TGFbeta signaling, controls Smad4 monoubiquitination. Cell.

[18] Miyazawa K, Miyazono K (2017). Regulation of TGF-beta family signaling by inhibitory Smads. Cold Spring Harb. Perspect. Biol..

[19] Ito Y (2001). Overexpression of Smad2 reveals its concerted action with Smad4 in regulating TGF-beta-mediated epidermal homeostasis. Dev. Biol..

[20] Ashcroft GS (1999). Mice lacking Smad3 show accelerated wound healing and an impaired local inflammatory response. Nat. Cell Biol..

[21] Zhang L, Du X, Wei S, Li D, Li Q (2016). A comprehensive transcriptomic view on the role of SMAD4 gene by RNAi-mediated knockdown in porcine follicular granulosa cells. Reproduction.

[22] Du X (2016). TGF-beta signaling controls FSHR signaling-reduced ovarian granulosa cell apoptosis through the SMAD4/miR-143 axis. Cell Death Dis..

[23] Liffers ST (2011). Keratin 23, a novel DPC4/Smad4 target gene which binds 14-3-3ε. BMC Cancer.

[24] Fei T (2010). Genome-wide mapping of SMAD target genes reveals the role of BMP signaling in embryonic stem cell fate determination. Genome Res..

[25] Takeda H (2016). Sleeping Beauty transposon mutagenesis identifies genes that cooperate with mutant Smad4 in gastric cancer development. Proc. Natl Acad. Sci. USA.

[26] Yu C, Zhang YL, Fan HY (2013). Selective Smad4 knockout in ovarian preovulatory follicles results in multiple defects in ovulation. Mol. Endocrinol..

[27] Liu J (2014). MicroRNA-26b functions as a proapoptotic factor in porcine follicular Granulosa cells by targeting Sma-and Mad-related protein 4. Biol. Reprod..

[28] Davis BN, Hilyard AC, Nguyen PH, Lagna G, Hata A (2010). Smad proteins bind a conserved RNA sequence to promote microRNA maturation by Drosha. Mol. Cell.

[29] Yao G (2010). MicroRNA-224 is involved in transforming growth factor-beta-mediated mouse granulosa cell proliferation and granulosa cell function by targeting Smad4. Mol. Endocrinol..

[30] Fu N (2016). Role of LncRNA-activated by transforming growth factor beta in the progression of hepatitis C virus-related liver fibrosis. Discov. Med..

[31] Mishra S (2014). Androgen receptor and microRNA-21 axis downregulates transforming growth factor beta receptor II (TGFBR2) expression in prostate cancer. Oncogene.

[32] Moisá SJ, Shike DW, Shoup L, Loor JJ (2016). Maternal plane of nutrition during late-gestation and weaning age alter steer calf longissimus muscle adipogenic microRNA and target gene expression. Lipids.

[33] Zhao N (2015). MicroRNA miR145 regulates TGFBR2 expression and matrix synthesis in vascular smooth muscle cells. Circ. Res..

[34] Bekenstein U (2017). Dynamic changes in murine forebrain miR-211 expression associate with cholinergic imbalances and epileptiform activity. Proc. Natl Acad. Sci. USA.

[35] Wang Y (2017). MiR-130a-3p attenuates activation and induces apoptosis of hepatic stellate cells in nonalcoholic fibrosing steatohepatitis by directly targeting TGFBR1 and TGFBR2. Cell Death Dis..

[36] Fedeli M (2016). miR-17 approximately 92 family clusters control iNKT cell ontogenesis via modulation of TGF-beta signaling. Proc. Natl Acad. Sci. USA.

[37] Liu L (2015). Enhanced expression of miR-425 promotes esophageal squamous cell carcinoma tumorigenesis by targeting SMAD2. J. Genet. Genomics.

[38] Cristobal I, Madoz-Gurpide J, Rojo F, Garcia-Foncillas J (2016). Potential therapeutic value of miR-425-5p in metastatic colorectal cancer. J. Cell. Mol. Med..

[39] He B (2016). CTNNA3 is a tumor suppressor in hepatocellular carcinomas and is inhibited by miR-425. Oncotarget.

[40] Wang Y (2016). Identification of a three-miRNA signature as a blood-borne diagnostic marker for early diagnosis of lung adenocarcinoma. Oncotarget.

[41] Ren RJ (2016). Peripheral blood microRNA expression profiles in Alzheimer’s disease: screening, validation, association with clinical phenotype and implications for molecular mechanism. Mol. Neurobiol..

[42] Lounis H (1998). Mapping of chromosome 3p deletions in human epithelial ovarian tumors. Oncogene.

[43] Yang S (2013). Expression patterns and regulatory functions of microRNAs during the initiation of primordial follicle development in the neonatal mouse ovary. Biol. Reprod..

