# Supplementary material for: SMAD4 feedback regulates the canonical TGF-β signaling pathway to control granulosa cell apoptosis
Source: Cell Death Dis. 2018 Feb 2;9(2):151. doi: 10.1038/s41419-017-0205-2 (PMC5833407; doi:10.1038/s41419-017-0205-2)

**Supplementary Figure Legends**

**Supplementary Figure 1.** DE mRNAs were identified in SMAD4-silenced porcine GCs. (**a**) Heat map for all 1025 differentially expressed mRNAs identified in GCs treated with SMAD4-siRNA compared with those treated with NC-siRNA by RNA-Seq. Color gradations indicate the expression level (a low level is indicated by green, and a high level is indicated by red). (**b**) Heat map for 147 SMAD4-induced mRNAs predicted to be the targets of SMAD4-inhibited miRNAs based on miRNA-mRNA pair analysis. Color gradations indicate the expression level (a low level is indicated by blue, and a high level is indicated by yellow). (**c**) Heat map for 65 SMAD4-inhibited mRNAs predicted to be the targets of SMAD4-induced miRNAs. Color gradations indicate the expression level (a low level is indicated by blue, and a high level is indicated by red).

**Supplementary Figure 2.** Multiple-sequence alignment of miRNA genes. (**a**) The multiple-sequence alignment of *pre-miR-425* from ten different species. (**b**) The multiple-sequence alignment of *pre-miR-130a* from six different species. (**c**) The multiple-sequence alignment of *pre-miR-1306* from five different species. (**d**) The multiple-sequence alignment of *pre-miR-143* from 11 different species. Conserved regions are indicated by black boxes. Mature sequences are indicated by asterisks. Seed sequences are underlined.

**Supplementary Figure 3.** Schematic diagram showing the miRNA response element (MRE) and mutations within the 3’UTR of the porcine *TGFBR2* mRNA. (**a**) ssc-miR-425, (**b**) ssc-miR-1306, (**c**) ssc-miR-130a, and (**d**) ssc-miR-143. The wild-type *TGFBR2* 3’UTR is indicated in red. The mutated *TGFBR2* 3’UTR is indicated in blue. Numbers in (**a-d**) indicate the nucleotides (nt) of the MRE in the 3’UTR of the porcine *TGFBR2* mRNA.

**Supplementary Figure 4.** Identification of the porcine miR-425 promoter. (**a**) Location of miR-425 and the host gene DALRD3 on the genome of the pig or other species deposited in NCBI and UCSC Genome databases (http://genome.ucsc.edu/). Potential promoters predicted in (**b**) are indicated by arrows. (**b**) Identification of the promoter elements related to the miR-425/DALRD3 genomic DNA sequence by in silico analyses (http://www.cbs.dtu.dk/services/Promoter/). Outputs represent the predicted transcription start site occurring within 250 base pairs around the indicated position. Three high-scored promoters were chosen for the following experiments. (**c**) Luciferase activity assay for the candidate promoter. Luciferase plasmids of three candidate promoters (prom1, prom2, and prom3) were constructed and transfected into HEK293 cells. (**d**) The effect of SMAD4 on the activity of the candidate promoters. Luciferase assays were performed to detect the luciferase activity of prom1, prom2, and prom3 in pGCs treated with SMAD4-siRNA (100 nM). (**e**) The effect of SMAD4 on the mRNA level of the host gene DALRD3 in pGCs. pGCs were transfected with SMAD4-siRNA, and the DALRD3 mRNA level was measured by qRT-PCR at different times*.* (**f**) The effect of SMAD4 on the pre-miR-425 mRNA level in pGCs. (**g**) The effect of SMAD4 on the mature miR-425 mRNA level in pGCs. Three independent experiments were performed in (**c-f**). *P*-values were calculated using the two tailed Student’s *t*-test. **, *P* < 0.01; ns, no significant difference.

**Supplementary Figure 5.** Identification of SBE motifs in the porcine miR-425 gene promoter. Nucleotide numbering is relative to +1 at the beginning site of pre-miR-425. The SBE motifs are underlined. Pre-miR-425 is indicated by a red box.

**Supplementary Figure 6.** miR-425 inhibits SMAD2 in porcine GCs. (**a**) The effect of miR-425 on the mRNA level of SMAD2 in pGC was determined by qRT-PCR. (**b**) Two miR-425 binding sites (BSs) were found in the 3’UTR of porcine SMAD2 gene. (**c-d**) Luciferase activity assays. HEK293 cells were co-transfected with miR-425 mimics and a construct carrying the SMAD2 3’-UTR, and luciferase activity was measured. Average results from three independent experiments are shown. Error bars ± S.E.M. **, *P* < 0.01; ns, no significant difference.

**Supplementary Figure 1**

**
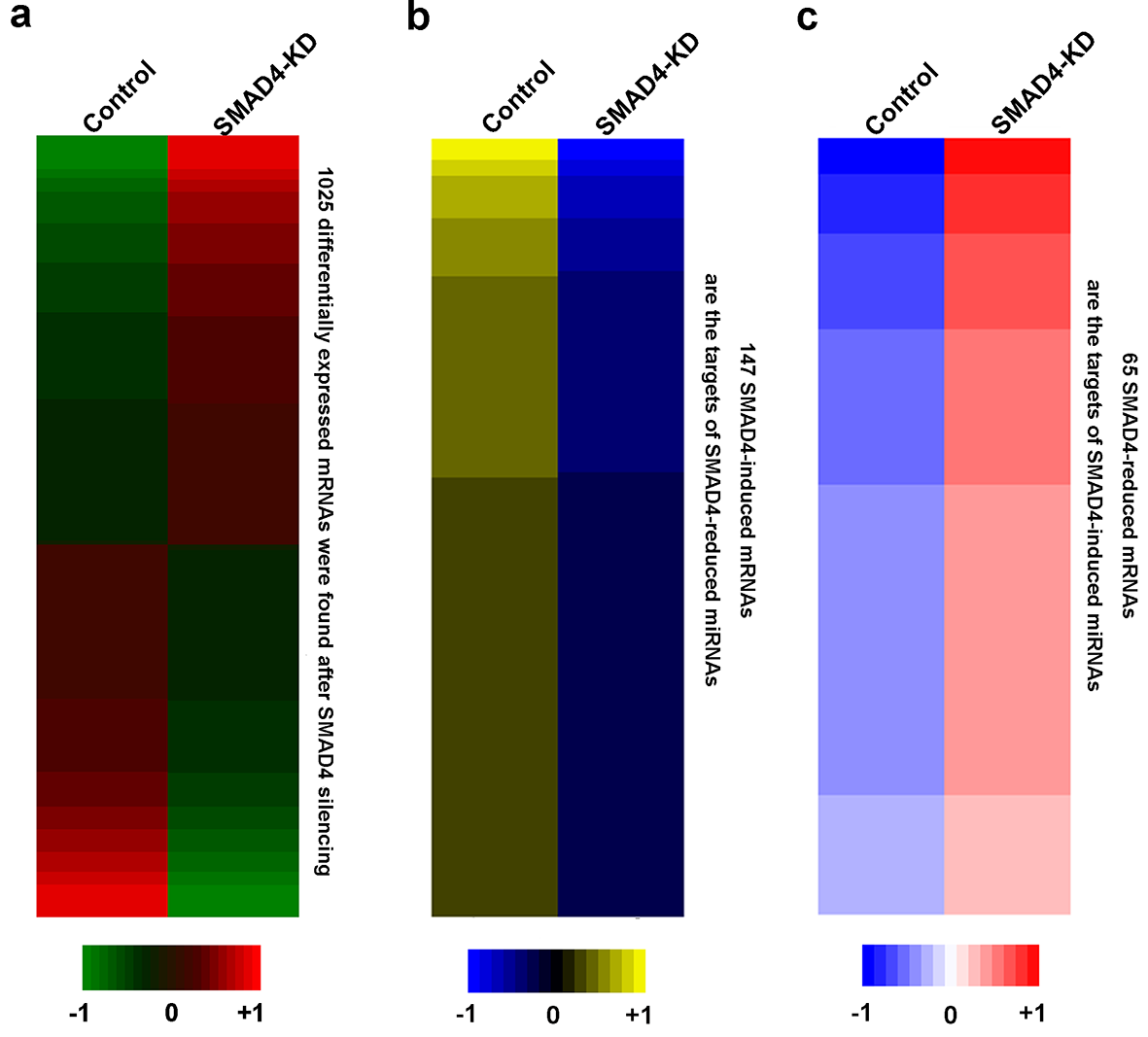
**

**Supplementary Figure 2**

**
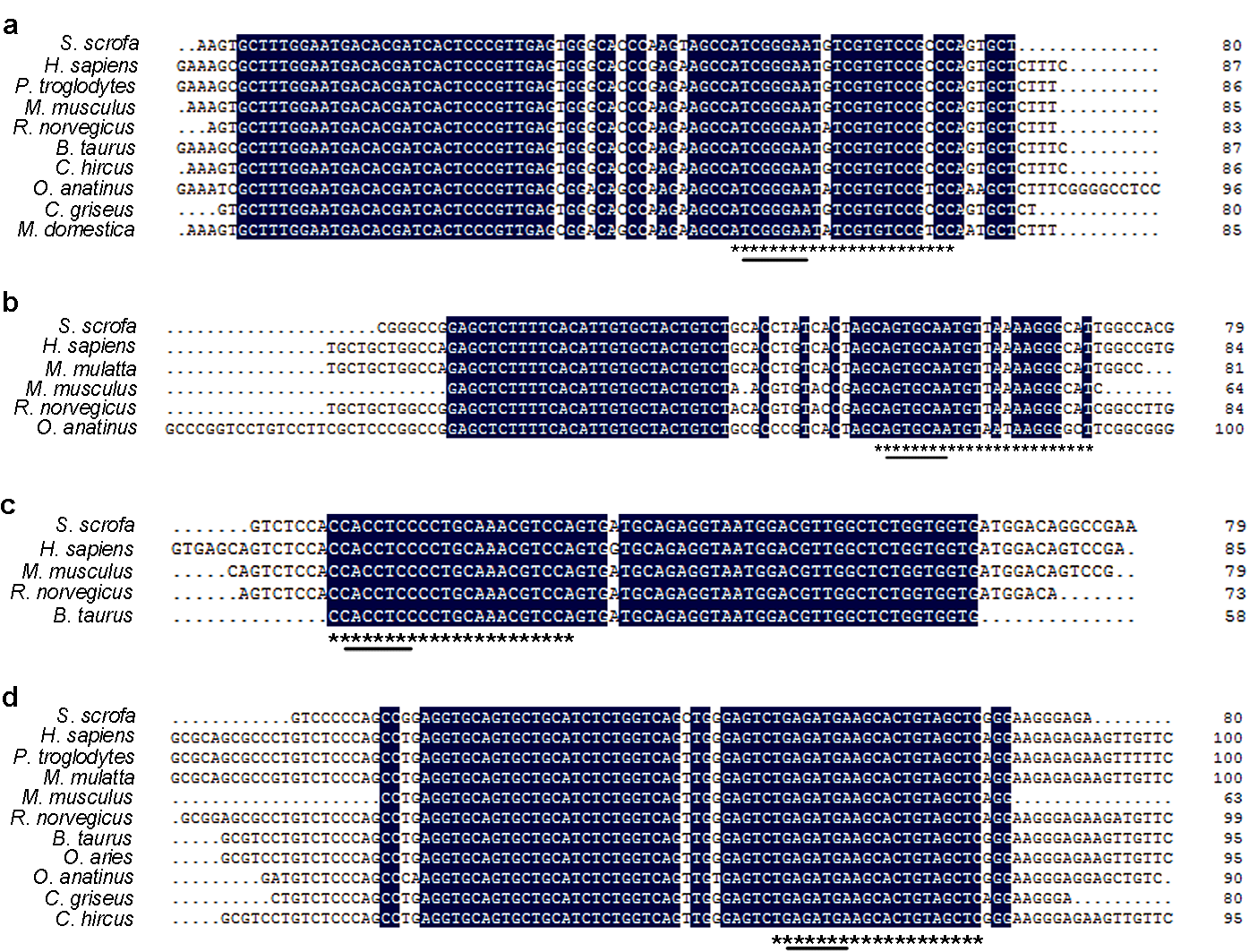
**

**Supplementary Figure 3**


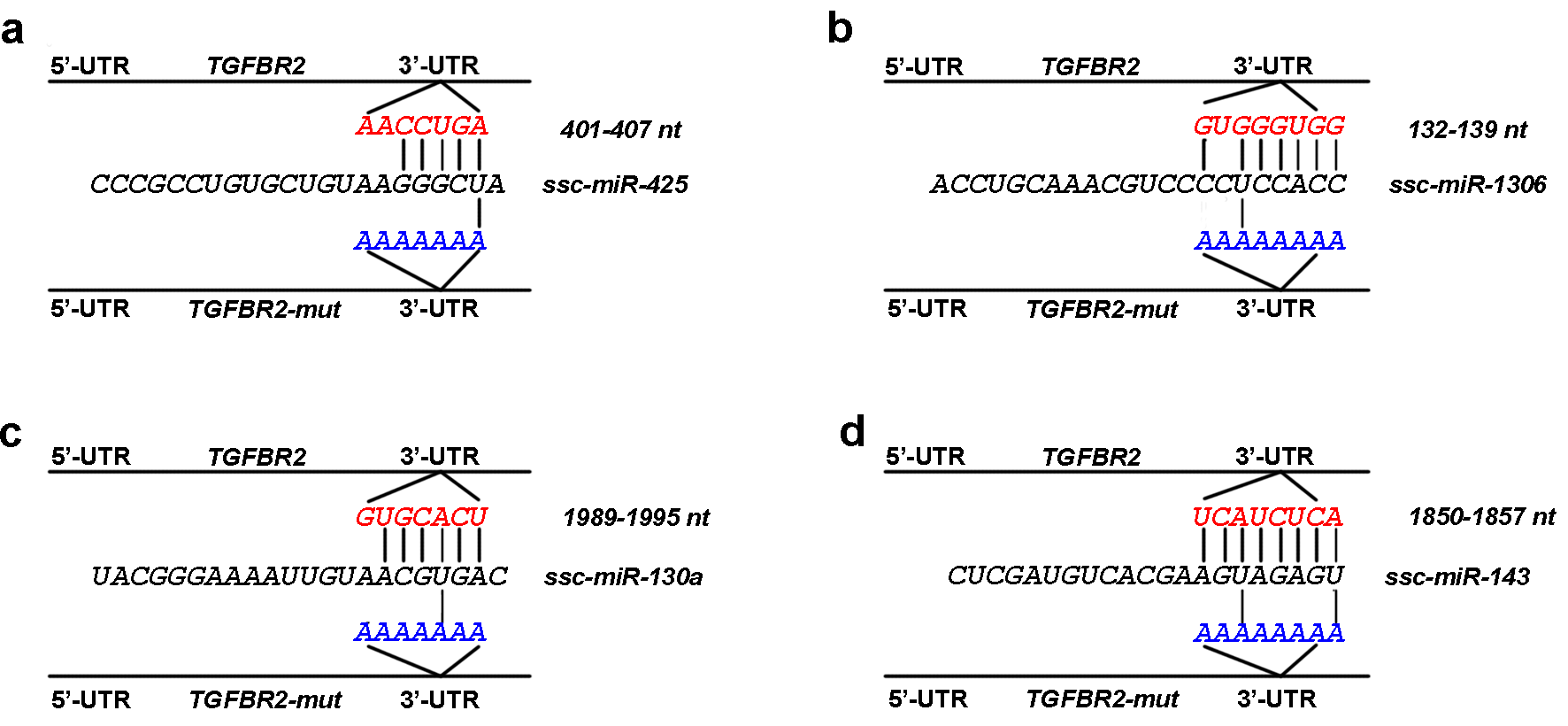


**Supplementary Figure 4**


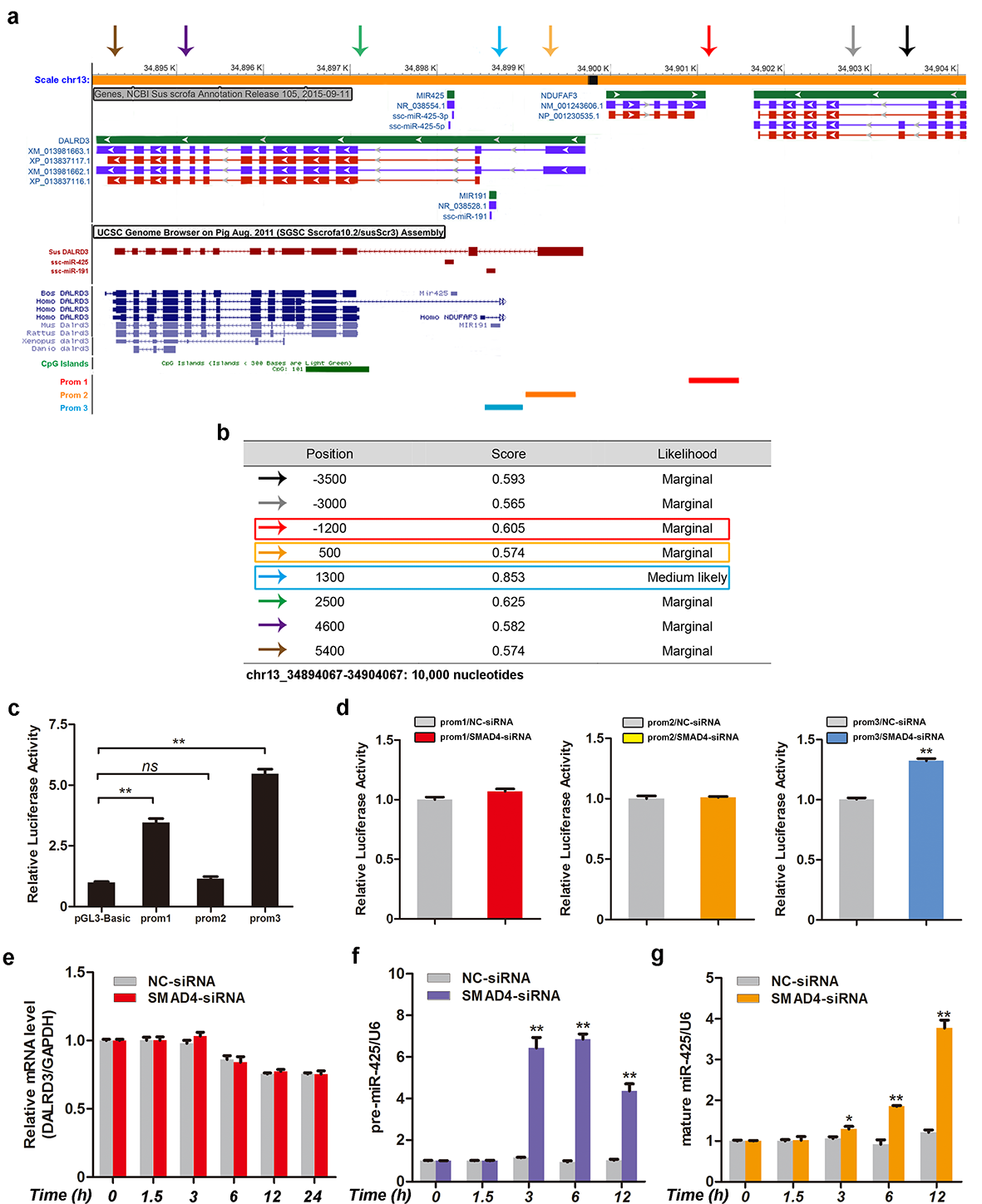


**Supplementary Figure 5**


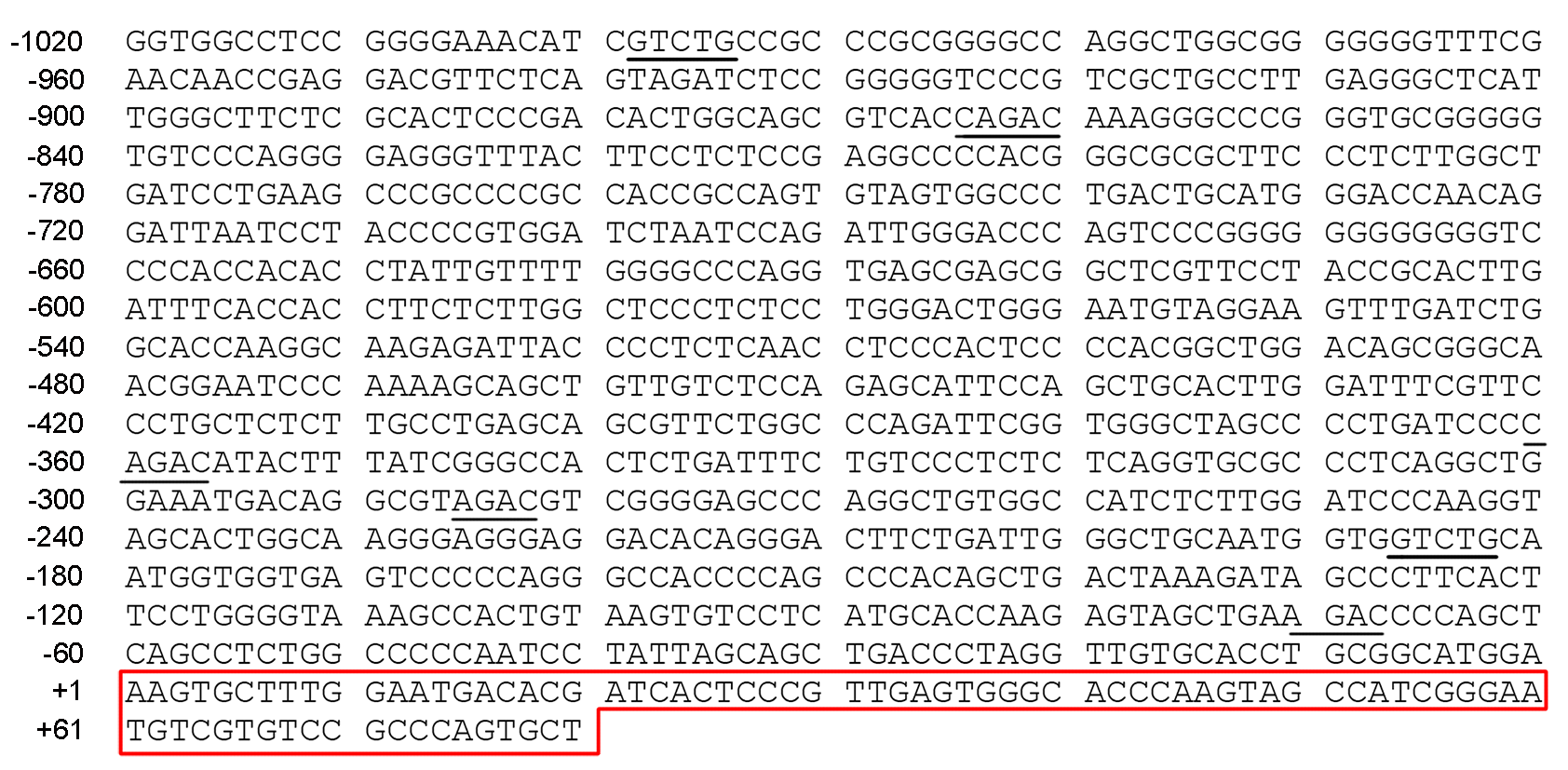


**Supplementary Figure 6**


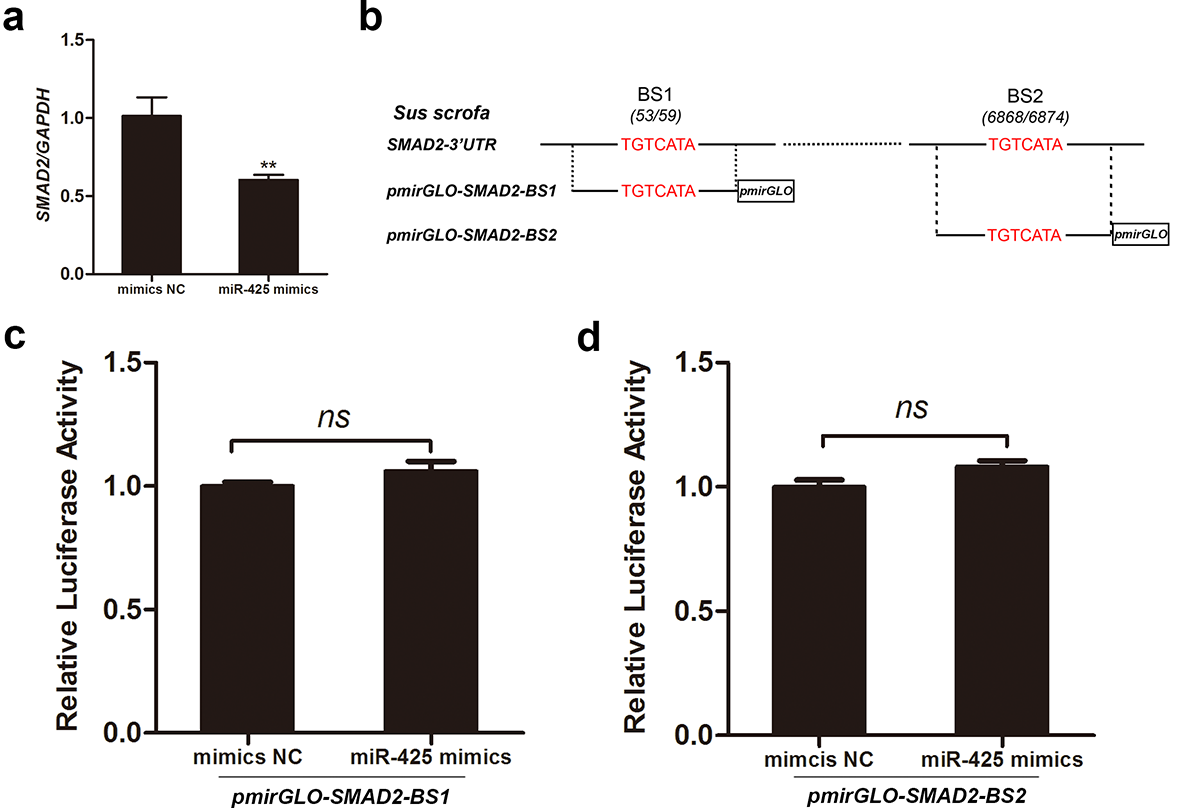

Supplement: Supplementary file 1 — Supplementary Figure [file 41419_2017_205_MOESM1_ESM.doc]
