# Supplementary material for: SMAD4 feedback regulates the canonical TGF-β signaling pathway to control granulosa cell apoptosis
Source: Cell Death Dis. 2018 Feb 2;9(2):151. doi: 10.1038/s41419-017-0205-2 (PMC5833407; doi:10.1038/s41419-017-0205-2)
Supplement: Supplementary file 2 — Supplementary Table [file 41419_2017_205_MOESM2_ESM.doc]

**Supplementary Table legends**

**Supplementary Table 1** The list of SMAD4-induced mRNAs that targeted by SMAD4-inhibited miRNAs.

**Supplementary Table 2** The list of SMAD4-reduced mRNAs that targeted by SMAD4-induced miRNAs.

**Supplementary Table 3** Oligonucleotide sequences used in this study.

**Supplementary Table 4** Primers designed for reverse-transcription and QRT-PCR.

**Supplementary Table 5** Primers used for plasmids construction and mutation.

**Supplementary Table 6** Primers for chromatin immunoprecipitation.

**Supplementary Table 1. The list of SMAD4-induced mRNAs that targeted by SMAD4-inhibited miRNAs**

| **Targetgene** | **GeneType** | **FoldChange** | **Log_2_FC** | **P-Value** | **FDR** | **Style** | **Control** | **SMAD4-KD** |
| --- | --- | --- | --- | --- | --- | --- | --- | --- |
| LOC100737304 | mRNA | 0.072653061 | -3.782832603 | 3.71E-09 | 6.08E-08 | down | 39.95716592 | 2.903010422 |
| LOC102163264 | mRNA | 0.100907029 | -3.308901415 | 3.50E-04 | 0.002264888 | down | 16.44286106 | 1.659200265 |
| LOC102159529 | mRNA | 0.129737609 | -2.946331335 | 0.002459647 | 0.012290245 | down | 13.24102448 | 1.717858861 |
| LOC733579 | mRNA | 0.131830151 | -2.923247722 | 9.33E-14 | 2.40E-12 | down | 80.815454 | 10.65391354 |
| LOC100524374 | mRNA | 0.163469388 | -2.612907601 | 9.83E-12 | 2.08E-10 | down | 76.64362248 | 12.52888604 |
| LOC102162371 | mRNA | 0.194159043 | -2.364689193 | 7.57E-10 | 1.34E-08 | down | 70.83536883 | 13.75332742 |
| GDF1 | mRNA | 0.201814059 | -2.308901415 | 0.008638767 | 0.035188101 | down | 13.30573625 | 2.68528464 |
| FAM188B | mRNA | 0.231871472 | -2.108602764 | 4.38E-08 | 6.27E-07 | down | 65.35765936 | 15.15457668 |
| LOC100738600 | mRNA | 0.234460451 | -2.092583508 | 1.31E-40 | 1.04E-38 | down | 392.3395489 | 91.98810753 |
| LOC100156608 | mRNA | 0.239313293 | -2.06302756 | 2.67E-08 | 3.94E-07 | down | 69.54919901 | 16.64404783 |
| LOC100736912 | mRNA | 0.251491366 | -1.991419225 | 1.84E-09 | 3.16E-08 | down | 85.41954136 | 21.48227712 |
| LOC102163654 | mRNA | 0.251491366 | -1.991419225 | 2.58E-05 | 2.21E-04 | down | 41.85312772 | 10.52570025 |
| SLA-DQA1 | mRNA | 0.259475219 | -1.946331335 | 0.001622416 | 0.008620291 | down | 24.27521154 | 6.298815823 |
| RUNX2 | mRNA | 0.300088731 | -1.736538951 | 2.13E-04 | 0.001466695 | down | 39.77023181 | 11.9345984 |
| NFYA | mRNA | 0.31264637 | -1.677396328 | 0.012063896 | 0.046189871 | down | 19.28512263 | 6.029423587 |
| SOX13 | mRNA | 0.340561224 | -1.554013912 | 0.012394071 | 0.047179962 | down | 21.62493355 | 7.364613849 |
| SLC29A4 | mRNA | 0.340561224 | -1.554013912 | 0.011599256 | 0.044770021 | down | 22.03128442 | 7.5030012 |
| FBLN7 | mRNA | 0.355830051 | -1.490739738 | 2.02E-06 | 2.21E-05 | down | 83.57321943 | 29.73786296 |
| MMP9 | mRNA | 0.384872164 | -1.377548762 | 2.51E-08 | 3.72E-07 | down | 131.2976503 | 50.53281087 |
| IGFBP5 | mRNA | 0.393199179 | -1.346667785 | 3.03E-33 | 1.92E-31 | down | 634.4592918 | 249.4688727 |
| LOC100513927 | mRNA | 0.396113765 | -1.33601326 | 1.09E-07 | 1.47E-06 | down | 125.7356875 | 49.80563654 |
| DFNB31 | mRNA | 0.407107671 | -1.29651769 | 0.002515602 | 0.012515617 | down | 42.87403903 | 17.45435016 |
| LOC100738607 | mRNA | 0.415020847 | -1.268744288 | 2.72E-09 | 4.56E-08 | down | 172.5753625 | 71.62237314 |
| SELP | mRNA | 0.417164427 | -1.261311956 | 3.89E-06 | 3.98E-05 | down | 105.0967659 | 43.84263211 |
| LOC100738037 | mRNA | 0.428239589 | -1.223509923 | 0.003721912 | 0.017563921 | down | 43.79031914 | 18.75274825 |
| LOC102160189 | mRNA | 0.430578284 | -1.215652536 | 0 | 0 | down | 14395.45941 | 6198.372206 |
| CRISPLD1 | mRNA | 0.431377551 | -1.212976995 | 2.38E-06 | 2.58E-05 | down | 117.6539796 | 50.75328561 |
| LOC102160244 | mRNA | 0.443642974 | -1.172528973 | 1.43E-07 | 1.89E-06 | down | 155.5335067 | 69.00134756 |
| LOC100521079 | mRNA | 0.445825603 | -1.165448625 | 3.05E-05 | 2.57E-04 | down | 98.7973666 | 44.04639554 |
| CALN1 | mRNA | 0.44861077 | -1.15646384 | 0.001153208 | 0.006379515 | down | 60.87364684 | 27.30857356 |
| TGFBR2 | mRNA | 0.564412211 | -0.825178894 | 9.52E-14 | 2.44E-12 | down | 614.6250279 | 346.9018711 |
| LDB3 | mRNA | 0.459866112 | -1.120714207 | 5.65E-04 | 0.003453408 | down | 72.56458017 | 33.36999135 |
| LOC100624895 | mRNA | 0.463348605 | -1.109830067 | 7.39E-04 | 0.004367115 | down | 70.79663066 | 32.80352004 |
| LOC100517759 | mRNA | 0.472244898 | -1.082392885 | 0.002424852 | 0.012147079 | down | 59.89315062 | 28.2842348 |
| LOC100739012 | mRNA | 0.475201709 | -1.073388072 | 2.33E-05 | 2.02E-04 | down | 118.4181538 | 56.272509 |
| LOC100524748 | mRNA | 0.478759982 | -1.062625527 | 0.010187402 | 0.040103478 | down | 44.50841185 | 21.30884647 |
| SORBS1 | mRNA | 0.480458642 | -1.057515846 | 5.41E-08 | 7.64E-07 | down | 201.1266105 | 96.63301814 |
| AFF2 | mRNA | 0.485072367 | -1.043728098 | 8.77E-04 | 0.005060555 | down | 77.21282329 | 37.45380697 |
| LOC100519213 | mRNA | 0.489997694 | -1.029153135 | 9.11E-17 | 2.88E-15 | down | 495.3335106 | 242.7122779 |
| LOC100153213 | mRNA | 0.490505293 | -1.02765939 | 0.002756306 | 0.013562045 | down | 64.37265918 | 31.57513005 |
| COLL11A1 | mRNA | 0.493352152 | -1.019310293 | 1.05E-45 | 9.49E-44 | down | 1468.961253 | 724.715195 |
| LOC100739819 | mRNA | 0.495361781 | -1.013445531 | 0.011324084 | 0.043866961 | down | 47.30928445 | 23.43521141 |
| PHKA2 | mRNA | 0.495520398 | -1.012983647 | 5.08E-08 | 7.20E-07 | down | 219.1611245 | 108.5988077 |
| TMOD1 | mRNA | 0.49819242 | -1.005225024 | 8.91E-06 | 8.52E-05 | down | 147.8415139 | 73.65352156 |
| AGXT2 | mRNA | 0.499201795 | -1.002304973 | 1.33E-08 | 2.04E-07 | down | 243.2314298 | 121.4215663 |
| MORC2 | mRNA | 0.500142531 | -0.999588802 | 8.06E-54 | 8.45E-52 | down | 1806.671011 | 903.5930116 |
| KLHL38 | mRNA | 0.506475667 | -0.981435136 | 0.013109319 | 0.04939997 | down | 48.286749 | 24.45606342 |
| LOC100522176 | mRNA | 0.508854125 | -0.974675961 | 2.79E-09 | 4.64E-08 | down | 280.8988764 | 142.9365521 |
| SMOC1 | mRNA | 0.512845138 | -0.963404849 | 0.007382418 | 0.030935527 | down | 58.3775171 | 29.93862582 |
| LOC100620265 | mRNA | 0.514054678 | -0.960006272 | 0.002539165 | 0.012619241 | down | 74.62476416 | 38.36120915 |
| LOC100516294 | mRNA | 0.518453358 | -0.947713889 | 0.001580344 | 0.008438867 | down | 83.83602829 | 43.46507042 |
| KLHL40 | mRNA | 0.520506044 | -0.942013179 | 4.56E-05 | 3.70E-04 | down | 141.2539421 | 73.52353061 |
| SLC25A43 | mRNA | 0.521206744 | -0.940072345 | 2.51E-04 | 0.001687362 | down | 114.3988341 | 59.6254438 |
| LOC100737680 | mRNA | 0.52288188 | -0.935443019 | 3.55E-07 | 4.43E-06 | down | 223.3653716 | 116.7937054 |
| LOC100516661 | mRNA | 0.523181012 | -0.934617915 | 1.02E-04 | 7.64E-04 | down | 130.2719427 | 68.15580675 |
| LOC100738528 | mRNA | 0.525778733 | -0.927472308 | 2.20E-05 | 1.92E-04 | down | 157.8016919 | 82.96877357 |
| P311 | mRNA | 0.526471458 | -0.925572775 | 2.14E-05 | 1.88E-04 | down | 158.8690367 | 83.64001338 |
| MRVI1 | mRNA | 0.53111893 | -0.912893143 | 2.72E-06 | 2.91E-05 | down | 198.9271652 | 105.6539832 |
| RASGRP3 | mRNA | 0.536268353 | -0.898972976 | 3.21E-07 | 4.03E-06 | down | 243.4456929 | 130.5522209 |
| LOC100156689 | mRNA | 0.540665404 | -0.88719205 | 3.14E-35 | 2.09E-33 | down | 1467.827434 | 793.6035131 |
| CAMK1D | mRNA | 0.544897959 | -0.875942007 | 0.006049419 | 0.026288855 | down | 73.98159708 | 40.31242126 |
| LOC102166027 | mRNA | 0.54538491 | -0.874653311 | 1.67E-06 | 1.87E-05 | down | 225.8696435 | 123.1858952 |
| LOC100511275 | mRNA | 0.545771193 | -0.873631847 | 1.78E-05 | 1.59E-04 | down | 181.732399 | 99.18430825 |
| MFSD4 | mRNA | 0.548354636 | -0.866818869 | 3.51E-05 | 2.92E-04 | down | 171.6728 | 94.13757573 |
| GFRA3 | mRNA | 0.554542171 | -0.850630919 | 0.013257508 | 0.049904042 | down | 63.89848623 | 35.43440527 |
| VCL | mRNA | 0.559949188 | -0.836632178 | 6.33E-78 | 8.95E-76 | down | 3761.486666 | 2106.241404 |
| CSGALNACT1 | mRNA | 0.560306385 | -0.835712163 | 2.21E-04 | 0.001515187 | down | 147.3629979 | 82.56842859 |
| RASL11B | mRNA | 0.560314711 | -0.835690725 | 1.53E-50 | 1.49E-48 | down | 2413.607097 | 1352.379563 |
| LOC100153940 | mRNA | 0.561766997 | -0.831956225 | 2.17E-20 | 8.72E-19 | down | 933.1730354 | 524.2258134 |
| KIDINS220 | mRNA | 0.561976506 | -0.831418277 | 9.26E-06 | 8.82E-05 | down | 214.5244209 | 120.5576845 |
| NEDD9 | mRNA | 0.563193481 | -0.828297459 | 2.39E-05 | 2.06E-04 | down | 196.3194861 | 110.5658548 |
| PRELP | mRNA | 0.563196212 | -0.828290465 | 1.74E-08 | 2.63E-07 | down | 349.2490137 | 196.6957214 |
| LOC100737606 | mRNA | 0.563291139 | -0.828047317 | 3.79E-04 | 0.002430073 | down | 138.9833689 | 78.28810021 |
| WBSCR17 | mRNA | 0.563687544 | -0.827032407 | 0.003097803 | 0.014981372 | down | 96.51023965 | 54.40161996 |
| LOC100152655 | mRNA | 0.56471243 | -0.824411707 | 0.001122489 | 0.006231999 | down | 117.844685 | 66.54835847 |
| RND1 | mRNA | 0.565472354 | -0.822471603 | 1.54E-58 | 1.82E-56 | down | 2902.73661 | 1641.417303 |
| ECE1 | mRNA | 0.566714006 | -0.819307236 | 5.29E-57 | 5.86E-55 | down | 2846.565658 | 1613.188628 |
| DNAJC8 | mRNA | 0.568334431 | -0.815187976 | 7.60E-06 | 7.39E-05 | down | 227.6566057 | 129.3850874 |
| NAV3 | mRNA | 0.568442933 | -0.814912574 | 0.001169822 | 0.006463677 | down | 119.8146866 | 68.10781185 |
| DESI2 | mRNA | 0.57068297 | -0.809238582 | 5.19E-09 | 8.42E-08 | down | 393.5185819 | 224.5743533 |
| C10H1orf115 | mRNA | 0.575876123 | -0.796169589 | 9.71E-17 | 3.07E-15 | down | 823.4898684 | 474.2281528 |
| LOC100737702 | mRNA | 0.580830706 | -0.78381037 | 0.003540074 | 0.016785943 | down | 104.8310345 | 60.88908383 |
| FZD4 | mRNA | 0.581787603 | -0.78143554 | 1.42E-10 | 2.69E-09 | down | 510.2263171 | 296.8433459 |
| ALDH1B1 | mRNA | 0.582808824 | -0.778905373 | 3.53E-06 | 3.67E-05 | down | 268.5585684 | 156.5183035 |
| LTBP2 | mRNA | 0.582879516 | -0.778730393 | 2.19E-80 | 3.23E-78 | down | 4502.555374 | 2624.447297 |
| ITGA8 | mRNA | 0.583700543 | -0.776699684 | 2.65E-22 | 1.17E-20 | down | 1185.033664 | 691.7047939 |
| LOC100739704 | mRNA | 0.589046451 | -0.763546689 | 7.86E-17 | 2.51E-15 | down | 904.0515122 | 532.5283346 |
| ACP2 | mRNA | 0.590306122 | -0.76046479 | 7.22E-04 | 0.004289729 | down | 150.0986362 | 88.60414395 |
| HERPUD2 | mRNA | 0.590853209 | -0.759128342 | 6.84E-04 | 0.004095618 | down | 151.9691911 | 89.79148422 |
| DDAH1 | mRNA | 0.593046402 | -0.753783105 | 3.50E-81 | 5.21E-79 | down | 4870.003563 | 2888.138089 |
| TTC17 | mRNA | 0.594204271 | -0.75096912 | 3.42E-04 | 0.002223966 | down | 172.891704 | 102.7329889 |
| LOC100737431 | mRNA | 0.595266678 | -0.748391958 | 1.08E-04 | 8.05E-04 | down | 203.5127324 | 121.1443482 |
| LOC100624161 | mRNA | 0.596228926 | -0.746061725 | 3.67E-06 | 3.78E-05 | down | 293.0010956 | 174.6957286 |
| ATP6V1A | mRNA | 0.598371559 | -0.740886489 | 1.78E-17 | 5.96E-16 | down | 1004.145304 | 600.8519913 |
| ITGA6 | mRNA | 0.599143207 | -0.739027219 | 8.03E-05 | 6.16E-04 | down | 216.9134046 | 129.9621928 |
| CHRDL1 | mRNA | 0.600700209 | -0.735282928 | 2.08E-19 | 7.83E-18 | down | 1144.413539 | 687.4494522 |
| CD34 | mRNA | 0.602255639 | -0.731552098 | 6.96E-08 | 9.68E-07 | down | 414.4572197 | 249.6091977 |
| BPI | mRNA | 0.602378762 | -0.731257189 | 1.26E-11 | 2.63E-10 | down | 654.5274998 | 394.273465 |
| SH3RF1 | mRNA | 0.602435014 | -0.731122472 | 1.60E-05 | 1.44E-04 | down | 265.7210984 | 160.0796936 |
| COL9A2 | mRNA | 0.604986957 | -0.725024055 | 1.90E-05 | 1.69E-04 | down | 265.7151534 | 160.7542021 |
| LOC100621687 | mRNA | 0.605442177 | -0.723938914 | 9.37E-04 | 0.005345281 | down | 159.5868905 | 96.6206344 |
| LOC100521067 | mRNA | 0.606034586 | -0.722527965 | 0.009404495 | 0.037598963 | down | 98.72030663 | 59.82792016 |
| PLA2G3 | mRNA | 0.60661506 | -0.72114678 | 1.45E-17 | 4.87E-16 | down | 1069.715972 | 648.9058183 |
| LOC100737020 | mRNA | 0.609556117 | -0.71416905 | 0 | 0 | down | 74713.68881 | 45542.18605 |
| FHL1C | mRNA | 0.611099846 | -0.710519978 | 2.06E-74 | 2.74E-72 | down | 5052.593832 | 3087.639311 |
| LOC100517718 | mRNA | 0.611477589 | -0.70962847 | 6.60E-48 | 6.21E-46 | down | 3216.76556 | 1966.98005 |
| ASAP1 | mRNA | 0.611489484 | -0.709600406 | 1.77E-05 | 1.59E-04 | down | 280.2402624 | 171.3639735 |
| B4GALT5 | mRNA | 0.611957697 | -0.708496168 | 6.10E-06 | 6.05E-05 | down | 312.3109459 | 191.1210872 |
| GADD45G | mRNA | 0.613408521 | -0.705079887 | 0.005321464 | 0.023546799 | down | 119.8221587 | 73.49993318 |
| AKAP2 | mRNA | 0.615247926 | -0.700760205 | 3.85E-10 | 7.05E-09 | down | 612.5927496 | 376.8964189 |
| PRKCB | mRNA | 0.61553288 | -0.700092172 | 0.009625262 | 0.038326598 | down | 105.0089258 | 64.63644648 |
| HSDL1 | mRNA | 0.615809337 | -0.699444352 | 0.002256822 | 0.011442003 | down | 146.4251209 | 90.16995671 |
| EPB41L1 | mRNA | 0.621947909 | -0.685134342 | 1.51E-12 | 3.48E-11 | down | 821.4159003 | 510.8779014 |
| COL8A1 | mRNA | 0.622575056 | -0.683680319 | 5.09E-09 | 8.26E-08 | down | 563.3695291 | 350.7398163 |
| SUSD1 | mRNA | 0.623856961 | -0.680712811 | 1.22E-04 | 8.97E-04 | down | 245.7181605 | 153.2929849 |
| MAMLD1 | mRNA | 0.624002244 | -0.680376879 | 0.006477371 | 0.027833994 | down | 123.5667692 | 77.10594119 |
| MRAS | mRNA | 0.627795237 | -0.671634012 | 1.59E-11 | 3.32E-10 | down | 778.8297036 | 488.9455782 |
| LUZP1 | mRNA | 0.630218033 | -0.66607706 | 1.45E-07 | 1.91E-06 | down | 483.0339805 | 304.4167249 |
| LOC100739215 | mRNA | 0.632628294 | -0.660570014 | 2.51E-04 | 0.0016876 | down | 238.4963155 | 150.8795172 |
| THSD4 | mRNA | 0.636443734 | -0.651895119 | 1.20E-06 | 1.38E-05 | down | 431.8352783 | 274.838857 |
| GDNF | mRNA | 0.636893719 | -0.650875452 | 0.013027729 | 0.049172839 | down | 113.3161152 | 72.17032196 |
| LOC100737374 | mRNA | 0.637566723 | -0.649351763 | 1.48E-18 | 5.34E-17 | down | 1427.718305 | 910.2656811 |
| LOC100152091 | mRNA | 0.637681825 | -0.649091331 | 4.47E-37 | 3.20E-35 | down | 2992.369749 | 1908.179804 |
| ADAM19 | mRNA | 0.639472903 | -0.645044867 | 5.43E-05 | 4.32E-04 | down | 305.5031575 | 195.3609911 |
| GAS6 | mRNA | 0.639605803 | -0.644745067 | 2.87E-34 | 1.88E-32 | down | 2796.260534 | 1788.504464 |
| LOC100512371 | mRNA | 0.640728684 | -0.642214516 | 3.70E-04 | 0.002378117 | down | 240.0341644 | 153.7967743 |
| CHI3L1 | mRNA | 0.643943597 | -0.634993767 | 1.47E-22 | 6.60E-21 | down | 1854.652838 | 1194.291819 |
| KIAA0513 | mRNA | 0.644976647 | -0.632681169 | 2.22E-13 | 5.50E-12 | down | 1053.370787 | 679.3995583 |
| EFCAB14 | mRNA | 0.645273899 | -0.632016424 | 4.89E-04 | 0.003035843 | down | 238.6094435 | 153.968446 |
| LOC100624854 | mRNA | 0.647208712 | -0.627697066 | 3.36E-06 | 3.51E-05 | down | 430.4907202 | 278.6173447 |
| USP13 | mRNA | 0.647624624 | -0.626770255 | 0.006685833 | 0.028561301 | down | 147.0800984 | 95.25269335 |
| VAMP2 | mRNA | 0.648409041 | -0.625023888 | 5.53E-04 | 0.003386494 | down | 239.9962839 | 155.6157603 |
| C4H1orf226 | mRNA | 0.648445546 | -0.624942666 | 0.006253731 | 0.027040892 | down | 150.4689851 | 97.57094327 |
| LOC100522001 | mRNA | 0.649227094 | -0.623204885 | 7.53E-86 | 1.26E-83 | down | 7806.418448 | 5068.138366 |
| ARX | mRNA | 0.650922761 | -0.619441732 | 7.76E-07 | 9.23E-06 | down | 501.3481227 | 326.3389045 |
| KREMEN1 | mRNA | 0.653131115 | -0.614555454 | 0.002024004 | 0.010433609 | down | 199.1642377 | 130.0803608 |
| LOC100524447 | mRNA | 0.654532083 | -0.611464185 | 2.79E-07 | 3.53E-06 | down | 558.0272981 | 365.2467698 |
| UNC119B | mRNA | 0.655673918 | -0.608949588 | 1.00E-11 | 2.11E-10 | down | 988.6119508 | 648.2070711 |
| SLC45A3 | mRNA | 0.656067888 | -0.608082987 | 2.40E-04 | 0.001624763 | down | 288.8682215 | 189.517164 |
| RBM33 | mRNA | 0.657906837 | -0.60404479 | 0.007475104 | 0.031248237 | down | 155.545731 | 102.3345999 |
| ANTXR1 | mRNA | 0.658054131 | -0.603721832 | 1.39E-07 | 1.84E-06 | down | 603.7711481 | 397.3140981 |
| ANO6 | mRNA | 0.658309735 | -0.603161562 | 1.39E-05 | 1.27E-04 | down | 411.9850187 | 271.2137487 |
| KCTD20 | mRNA | 0.65984648 | -0.599797688 | 0.004162735 | 0.019340857 | down | 181.4053676 | 119.6996933 |
| IARS2 | mRNA | 0.661089436 | -0.597082634 | 3.20E-04 | 0.002097088 | down | 289.0004516 | 191.0551455 |
| SLC25A13 | mRNA | 0.66124885 | -0.596734787 | 2.73E-09 | 4.57E-08 | down | 790.5359358 | 522.7409782 |
| KDSR | mRNA | 0.66198689 | -0.595125448 | 0.002354874 | 0.011847985 | down | 208.0381387 | 137.7185206 |
| SVEP1 | mRNA | 0.662656697 | -0.593666449 | 6.72E-11 | 1.32E-09 | down | 963.4283422 | 638.4222431 |

The information of TGFBR2 was shown within red letters.

**Supplementary Table 2. The list of SMAD4-reduced mRNAs that targeted by SMAD4-induced miRNAs**

| **Targetgene** | **GeneType** | **FoldChange** | **Log_2_FC** | **P-Value** | **FDR** | **Style** | **Control** | **SMAD4-KD** |
| --- | --- | --- | --- | --- | --- | --- | --- | --- |
| LOC100519082 | mRNA | 7.240849302 | 2.856158925 | 0 | 0 | up | 391.6412369 | 2835.815177 |
| TEKT1 | mRNA | 6.659863946 | 2.735492705 | 6.31E-04 | 0.003811931 | up | 2.311924909 | 15.39710534 |
| LOC102167550 | mRNA | 5.059766764 | 2.339070884 | 1.49E-08 | 2.26E-07 | up | 9.819186691 | 49.68279447 |
| BBS1 | mRNA | 4.654336735 | 2.218575591 | 0.00934346 | 0.037441468 | up | 2.38492015 | 11.10022146 |
| LOC100738712 | mRNA | 4.630143861 | 2.211057019 | 1.27E-39 | 9.58E-38 | up | 61.80281861 | 286.1559412 |
| IFIT2 | mRNA | 4.359183673 | 2.124057993 | 4.25E-08 | 6.10E-07 | up | 11.89745347 | 51.86318492 |
| BCL2A1 | mRNA | 4.238095238 | 2.083416008 | 0.002601859 | 0.012898397 | up | 3.778908651 | 16.01537476 |
| LOC100525698 | mRNA | 4.17755102 | 2.062657448 | 2.11E-07 | 2.73E-06 | up | 11.52011797 | 48.12588056 |
| LOC102162677 | mRNA | 3.519132653 | 1.815219897 | 3.51E-20 | 1.38E-18 | up | 50.21655891 | 176.7187322 |
| DPF1 | mRNA | 3.360204082 | 1.748548858 | 0.00772177 | 0.032124142 | up | 4.620047309 | 15.52430183 |
| ETV7 | mRNA | 3.027210884 | 1.597989181 | 4.57E-04 | 0.002855364 | up | 9.967826073 | 30.17471158 |
| MDFI | mRNA | 2.983965015 | 1.577230621 | 6.77E-04 | 0.004060708 | up | 9.674254046 | 28.86763562 |
| PAQR7 | mRNA | 2.96543107 | 1.568241838 | 5.07E-08 | 7.19E-07 | up | 25.20887352 | 74.7551768 |
| DTX3 | mRNA | 2.906122449 | 1.539095492 | 8.89E-09 | 1.40E-07 | up | 29.36956139 | 85.35154166 |
| HERC5 | mRNA | 2.7610251 | 1.465204003 | 2.56E-29 | 1.44E-27 | up | 126.1326568 | 348.2554312 |
| LOC100739645 | mRNA | 2.667729592 | 1.415612439 | 1.03E-04 | 7.65E-04 | up | 16.32836339 | 43.5596582 |
| KDM4B | mRNA | 2.392234943 | 1.258359084 | 1.34E-20 | 5.46E-19 | up | 122.8464419 | 293.877551 |
| FAM198B | mRNA | 2.371028525 | 1.24551302 | 4.08E-13 | 9.90E-12 | up | 76.38192699 | 181.1037277 |
| TWIST2 | mRNA | 2.36122449 | 1.23953521 | 0.001025619 | 0.005789395 | up | 15.82528881 | 37.3670595 |
| CDT1 | mRNA | 2.333979592 | 1.222791946 | 1.16E-08 | 1.79E-07 | up | 49.28050463 | 115.0196921 |
| LOC100621293 | mRNA | 2.297118848 | 1.1998255 | 2.96E-08 | 4.32E-07 | up | 48.54161015 | 111.5058476 |
| LOC100157002 | mRNA | 2.288571429 | 1.194447321 | 2.10E-07 | 2.72E-06 | up | 42.96732335 | 98.33378858 |
| MXD3 | mRNA | 2.270408163 | 1.182951682 | 1.62E-06 | 1.81E-05 | up | 37.4962827 | 85.13186633 |
| MFAP2 | mRNA | 2.270408163 | 1.182951682 | 0.003012599 | 0.014630662 | up | 14.34377241 | 32.56621798 |
| RPP25 | mRNA | 2.249954036 | 1.169895529 | 2.08E-12 | 4.72E-11 | up | 82.54076845 | 185.7129351 |
| INCENP | mRNA | 2.182302772 | 1.125851275 | 2.50E-07 | 3.18E-06 | up | 48.38104041 | 105.5820786 |
| R3HCC1L | mRNA | 2.169501134 | 1.11736334 | 8.64E-06 | 8.30E-05 | up | 36.59478728 | 79.39243249 |
| E2F1 | mRNA | 2.106073858 | 1.074556031 | 4.87E-13 | 1.17E-11 | up | 105.2431116 | 221.6497661 |
| LOC100525721 | mRNA | 2.092224231 | 1.065037479 | 3.40E-08 | 4.91E-07 | up | 62.55394288 | 130.8768751 |
| RNF138 | mRNA | 2.061776062 | 1.043887644 | 2.95E-04 | 0.001952968 | up | 28.08988764 | 57.91505792 |
| OAS2 | mRNA | 2.027409711 | 1.019637667 | 1.15E-56 | 1.24E-54 | up | 567.524338 | 1150.604354 |
| LOC100737768 | mRNA | 2.025902669 | 1.018564864 | 4.73E-06 | 4.78E-05 | up | 47.3476226 | 95.92167499 |
| C1H6orf211 | mRNA | 2.012204882 | 1.008777207 | 0.001346915 | 0.007319457 | up | 23.71828261 | 47.72604406 |
| LOC100515724 | mRNA | 1.978636561 | 0.98450664 | 9.54E-10 | 1.68E-08 | up | 90.95588833 | 179.9686461 |
| CCDC97 | mRNA | 1.977777778 | 0.983880335 | 0.012348354 | 0.047031803 | up | 15.23863706 | 30.13863773 |
| IL15RA | mRNA | 1.888023631 | 0.916876822 | 0.002647047 | 0.013098956 | up | 25.52099774 | 48.1842468 |
| CCNF | mRNA | 1.860762654 | 0.895894047 | 1.11E-08 | 1.73E-07 | up | 96.71380109 | 179.9614292 |
| LOC100738277 | mRNA | 1.85581189 | 0.892050482 | 0.003139471 | 0.015145842 | up | 26.09055692 | 48.41916575 |
| PRPF40A | mRNA | 1.832259219 | 0.873623624 | 0.002503093 | 0.012471314 | up | 28.53416967 | 52.28199544 |
| LOC102157879 | mRNA | 1.830298273 | 0.872078775 | 0.003281257 | 0.015731165 | up | 27.08965425 | 49.5821474 |
| KLF16 | mRNA | 1.760724698 | 0.816169351 | 2.48E-04 | 0.001672895 | up | 48.18921643 | 84.84794355 |
| LOC100517400 | mRNA | 1.744629431 | 0.802920632 | 3.09E-04 | 0.002028932 | up | 48.28114875 | 84.23271306 |
| MX1 | mRNA | 1.736829213 | 0.796455897 | 5.78E-105 | 1.14E-102 | up | 1784.941396 | 3100.13836 |
| ATXN3 | mRNA | 1.732817265 | 0.793119522 | 8.67E-07 | 1.03E-05 | up | 92.0459595 | 159.4988278 |
| BATF2 | mRNA | 1.713791968 | 0.777191996 | 0.004471389 | 0.020465773 | up | 32.01421018 | 54.86569628 |
| NEK2 | mRNA | 1.712864893 | 0.776411359 | 0.001128768 | 0.006264348 | up | 42.08821477 | 72.09142547 |
| RHCG | mRNA | 1.711887755 | 0.77558811 | 1.48E-06 | 1.68E-05 | up | 92.17354435 | 157.7907619 |
| UBE2D3 | mRNA | 1.710565745 | 0.774473555 | 1.57E-12 | 3.61E-11 | up | 199.2906263 | 340.8997187 |
| TMEM106A | mRNA | 1.707514342 | 0.771897696 | 5.48E-07 | 6.67E-06 | up | 100.752159 | 172.0357565 |
| EEF1E1 | mRNA | 1.699144174 | 0.764808272 | 4.05E-05 | 3.32E-04 | up | 68.92769263 | 117.1180873 |
| UBE2T | mRNA | 1.696943566 | 0.762938587 | 3.81E-13 | 9.26E-12 | up | 216.7806546 | 367.8645371 |
| FAM174A | mRNA | 1.69433445 | 0.760718681 | 7.89E-04 | 0.004621851 | up | 46.58471465 | 78.93008689 |
| NFKBID | mRNA | 1.68886502 | 0.756054027 | 0.008138663 | 0.033476146 | up | 29.3111871 | 49.50263858 |
| CDH24 | mRNA | 1.687812861 | 0.755154952 | 0.003234134 | 0.015532145 | up | 36.37786304 | 61.3990251 |
| ADAMTS5 | mRNA | 1.685341444 | 0.753040906 | 0.002352121 | 0.011838439 | up | 39.03588862 | 65.78880091 |
| KLF2 | mRNA | 1.659746657 | 0.730963046 | 6.68E-04 | 0.004011821 | up | 51.8032904 | 85.98033808 |
| USP18 | mRNA | 1.618695146 | 0.694831304 | 7.66E-42 | 6.28E-40 | up | 907.8812967 | 1469.583049 |
| EMC10 | mRNA | 1.607479123 | 0.6848 | 1.24E-58 | 1.48E-56 | up | 1325.314833 | 2130.415925 |
| PUSL1 | mRNA | 1.564666831 | 0.645855492 | 0.001121019 | 0.006226332 | up | 60.43968047 | 94.56796328 |
| BIK | mRNA | 1.551445578 | 0.633613091 | 2.04E-04 | 0.001410467 | up | 81.46916053 | 126.3949689 |
| MFSD2A | mRNA | 1.528933092 | 0.612525274 | 3.17E-04 | 0.002073504 | up | 81.65965496 | 124.8521488 |
| ATF5 | mRNA | 1.522872414 | 0.606795078 | 1.52E-06 | 1.71E-05 | up | 148.1996764 | 225.689199 |
| STAM2 | mRNA | 1.520408163 | 0.604458676 | 0.003933221 | 0.01835459 | up | 53.67686527 | 81.61074414 |
| FGFR4 | mRNA | 1.513605442 | 0.597989181 | 1.74E-04 | 0.001231417 | up | 92.8326771 | 140.5120453 |
| C14H10orf54 | mRNA | 1.501398947 | 0.586307376 | 0.003215965 | 0.015455616 | up | 59.33809314 | 89.09015054 |

**Supplementary Table 3. Oligonucleotide sequences used in this study**

| **Name** | **Sequence (5’ to 3’)** | **Source** |
| --- | --- | --- |
| Mimics NC | UUGUACUACACAAAAGUACUG | This study |
| MiR-1306 | CCACCUCCCCUGCAAACGUCCA | This study |
| MiR-130a | CAGUGCAAUGUUAAAAGGGCAU | This study |
| MiR-143 | UGAGAUGAAGCACUGUAGCUC | Ref. 22 |
| MiR-425 | AUCGGGAAUGUCGUGUCCGCCC | This study |
| Inhibitor NC | CAGUACUUUUGUGUAGUACAA | This study |
| MiR-425 inhibitor | GGGCGGACACGACAUUCCCGAU | This study |
| NC-siRNA | UUCUCCGAACGUGUCACGUTT | This study |
|  | ACGUGACACGUUCGGAGAATT | This study |
| TGFBR2-siRNA | GCCAACAACAUCAACCACATT | This study |
|  | UGUGGUUGAUGUUGUUGGCTT | This study |
| SMAD4-siRNA | CACCAGGAAUUGAUCUCUCAGGAUU | Ref 28 |
|  | AAUCCUGAGAGAUCAAUUCCUGGUG |  |

**Supplementary Table 4. Primers designed for reverse-transcription and qPCR**

| **Genes** | **Primer sequence (5’ to 3’)** | **Usage** |
| --- | --- | --- |
| Mature-miR-1306 | CCTGTTGTCTCCAGCCACAAAAGAGCACAATATTTCAGGAGACAACAGGGGACGTT | Reverse-transcription^1^ |
| Mature-miR-130a | CCTGTTGTCTCCAGCCACAAAAGAGCACAATATTTCAGGAGACAACAGGATGCCCT | Reverse-transcription^1^ |
| Mature-miR-143 | CCTGTTGTCTCCAGCCACAAAAGAGCACAATATTTCAGGAGACAACAGGTGAGATG | Reverse-transcription^1^ |
| Mature-miR-425 | CCTGTTGTCTCCAGCCACAAAAGAGCACAATATTTCAGGAGACAACAGGGGGCGGA | Reverse-transcription^1^ |
| Mature-miR-29c | CCTGTTGTCTCCAGCCACAAAAGAGCACAATATTTCAGGAGACAACAGGGAACACC | Reverse-transcription^1^ |
| Mature-miR-423 | CCTGTTGTCTCCAGCCACAAAAGAGCACAATATTTCAGGAGACAACAGGAAAGTCT | Reverse-transcription^1^ |
| Mature-miR-10b | CCTGTTGTCTCCAGCCACAAAAGAGCACAATATTTCAGGAGACAACAGGCACAAAT | Reverse-transcription^1^ |
| Mature-miR-296 | CCTGTTGTCTCCAGCCACAAAAGAGCACAATATTTCAGGAGACAACAGGACAGGAT | Reverse-transcription^1^ |
| Mature-miR-100 | CCTGTTGTCTCCAGCCACAAAAGAGCACAATATTTCAGGAGACAACAGGCACAAGT | Reverse-transcription^1^ |
| Mature-miR-191 | CCTGTTGTCTCCAGCCACAAAAGAGCACAATATTTCAGGAGACAACAGGCAGCTGC | Reverse-transcription^1^ |
| Mature-miR-155 | CCTGTTGTCTCCAGCCACAAAAGAGCACAATATTTCAGGAGACAACAGGACCCCTA | Reverse-transcription^1^ |
| Mature-miR-186 | CCTGTTGTCTCCAGCCACAAAAGAGCACAATATTTCAGGAGACAACAGGAGCCCAA | Reverse-transcription^1^ |
| Mature-miR-935 | CCTGTTGTCTCCAGCCACAAAAGAGCACAATATTTCAGGAGACAACAGGGCGGTAG | Reverse-transcription^1^ |
| Mature-miR-27a | CCTGTTGTCTCCAGCCACAAAAGAGCACAATATTTCAGGAGACAACAGGTGCTCAC | Reverse-transcription^1^ |
| Mature-miR-1306 | F: CGGGCCACCACCTCCCCTGCAA | qPCR |
| Mature-miR-130a | F: CGGGCCAGTGCAATGTTAAA | qPCR |
| Mature-miR-143 | F: CGGGCGAGCTACAGTGCTT | qPCR |
| Mature-miR-425 | F: CGGGCATCGGGAATGTCGTG | qPCR |
| Mature-miR-29c | F: CGGGCTGACCGATTTCTCCT | qPCR |
| Mature-miR-423 | F: CGGGCTGAGGGGCAGAGAGCG | qPCR |
| Mature-miR-10b | F: CGGGCTACCCTGTAGAACCGA | qPCR |
| Mature-miR-296 | F: CGGGCAGGGCCCCCCCTCA | qPCR |
| Mature-miR-100 | F: CGGGCAACCCGTAGATCCGA | qPCR |
| Mature-miR-191 | F: CGGGCCAACGGAATCCCAAAA | qPCR |
| Mature-miR-155 | F: CGGGCTTAATGCTAATCGTGA | qPCR |
| Mature-miR-186 | F: CGGGCCAAAGAATTCTCCTT | qPCR |
| Mature-miR-935 | F: CGGGCCCAGTTACCGCTTCCG | qPCR |
| Mature-miR-27a | F: CGGGCAGGGCTTAGCTGCTT | qPCR |
| Common reverse primer for miRNAs | R: GCCACAAAAGAGCACAAT | qPCR |
| Pre-miR-1306 | F: GTCTCCACCACCTCCCCTGCAA | qPCR |
|  | R: GTTCGGCCTGTCCATCACCACC |  |
| Pre -miR-130a | F: CGGGCCGGAGCTCTTTTCACAT | qPCR |
|  | R: CGTGGCCAATGCCCTTTTAACA |  |
| Pre -miR-143 | F: GTCCCCCAGCCGGAGGTGCAGT | qPCR |
|  | R: TCTCCCTTCCCGAGCTACAGTGC |  |
| Pre -miR-425 | F: AAGTGCTTTGGAATGACACGAT | qPCR |
|  | R: AGCACTGGGCGGACACGACATT |  |
| Pre -miR-29c | F: ATCTCTTACACAGGCTGACCGA | qPCR |
|  | R: TCCCCCTACATCATAACCGATT |  |
| Pre -miR-423 | F: AAGTTAGGCTGAGGGGCAGAGA | qPCR |
|  | R: GTAGGAAGCAAGACTGAGGGGC |  |
| Pre -miR-10b | F: GTCTATATATACCCTGTAGAAC | qPCR |
|  | R: TCGACCATATATTCCCCTAGAA |  |
| Pre -miR-296 | F: GACCCTTCCGGAGGGCCCCCCC | qPCR |
|  | R: CAGAGCCTTCAGGAAAGCCTC |  |
| Pre -miR-100 | F: AGGCCTGTTGCCACAAACCCGT | qPCR |
|  | R: CACAGACACATACCTATAGA |  |
| Pre -miR-191 | F: ACAGCGGGCAACGGAATCCCAA | qPCR |
|  | R: AGAGAGCAGGGAACGAAATCCTT |  |
| Pre -miR-155 | F: CTATATGCTGTTAAUGCTAATT | qPCR |
|  | R: CATCATACCCTGTTAATGCTAA |  |
| Pre -miR-186 | F: TGCTTATAACTTTCCAAAGAAT | qPCR |
|  | R: CAAACTTCCCAAAAAATTCTCC |  |
| Pre -miR-935 | F: GGCGCAGGCGGCAGTGGCGGGA | qPCR |
|  | R: GAGCGGCGGCGGTAGCGGAAGC |  |
| Pre -miR-27a | F: TGGCCTGGGGAGCAGGGCTTAG | qPCR |
|  | R: TCCAGGGGGCGGAACTTAGCCA |  |
| DALRD3 | F: GTGCCCAAGCAGGTCATTCA | qPCR |
|  | R: CTCCTCAGGGCTTCGGTCA |  |
| SMAD2 | F: GAAGAGAAGTGGTGTGAGAAAGCAG | qPCR |
|  | R: AATACTGGAGGCAAAACTGGTGTC |  |
| GAPDH | F: GATGGTGAAGGTCGGAGTG | qPCR |
|  | R: CGAAGTTGTCATGGATGACC |  |

^1^. Stem-loop reverse-transcriptional primers designed for detect the expression level of mature miRNAs.

**Supplementary Table 5. Primers used for plasmids construction and mutation**

| **Plasmids** | **Primer sequence (5’ to 3’)** | **Vector** | **Usage** |
| --- | --- | --- | --- |
| Wild-TGFBR2 (1306/130a) | F: CTAGCTAGCTGGCCAAAGAACAGAGGCA | pmirGLO | 3’UTR vector construction |
|  | R: CCGCTCGAGCCAAAGACAAAGCCTCCCTC |  |
| Wild-TGFBR2 (425/143) | F: CTAGCTAGCAGCGTGACTGGCAGTGATC | pmirGLO | 3’UTR vector construction |
|  | R: CCGCTCGAGAACATAAAGCAAGCCACAG |  |
| Mut-TGFBR2 (1306) | F: CTGTCACCCCTTCtttttCACCCTGCTCACAGCAGGGG | pmirGLO | Mutation |
|  | R: GAAGCATCTTAAGCCTTTGACATTGTCATAGGATAAG |  |
| Mut-TGFBR2 (130a) | F: GCATCAACAGCCCTAAttttttCAGCATTTCTGGGCAC | pmirGLO | Mutation |
|  | R: CATCCCAATAGCTATTGCTCACTGACCTCTAGTGGTGA |  |
| Mut-TGFBR2 (143) | F: TCTGCTAAAACCCCAAtttttttAGACCTTCCAAAACCC | pmirGLO | Mutation |
|  | R: CATGCTGAAACTTGGGATTAGGTGGAACTTTTTGGAAC |  |
| Mut-TGFBR2 (425) | F: GCCCTTCCTTCTGCGTttttttCTGAGCCTGCCCATCA | pmirGLO | Mutation |
|  | R: TCTGTGACCGCCTCAGCACTTGGCAATCATGTGTGTGC |  |
| pmirGLO-SMAD2-BS1 | F: CTAGCTAGCCACTCCTTGCTGGATTGAA | pmirGLO | 3’UTR vector  construction |
|  | R: CCGCTCGAGTGCCCAACAAGACATCAGT |  |
| pmirGLO-SMAD2-BS2 | F: CTAGCTAGCTGCTCAAGAAGCCAGAATG | pmirGLO | 3’UTR vector  construction |
|  | R: CCGCTCGAGGACCACCACAGGGAAACAG |  |
| pGL3-SBE1 | F: GGGGTACCCGGAGTATCGCCCTATTCTTG | pGL3-Basic | Promoter vector  construction |
|  | R: CCGCTCGAGTCAAGTGCGGTAGGAACGAG |  |
| pGL3-SBE2 | F: GGGGTACCCGGGTTTCGAACAACCGAGGACGT | pGL3-Basic | Promoter vector  construction |
|  | R: CCGCTCGAGGGAAGTAAACCCTCCCCTGGGA |  |
| pGL3-SBE3 | F: GGGGTACCCGCACTTGGATTTCGTTCCCTGCTCT | pGL3-Basic | Promoter vector  construction |
|  | R: CCGCTCGAGGGGACAGAAATCAGAGTGGCCCGA |  |
| pGL3-SBE4 | F: GGGGTACCCCGGGCCACTCTGATTTCTGTCCCT | pGL3-Basic | Promoter vector  construction |
|  | R: CCGCTCGAGCCTCCCTCCCTTGCCAGTGCT |  |
| pGL3-SBE5 | F: CGGGGTACCCTCGTTCCTACCGCACTTGA | pGL3-Basic | Promoter vector  construction |
|  | R: CCGCTCGAGCACTGGGCGGACACGACA |  |
| pGL3-SBE6 | F: GGGGTACCCGCCCTTCACTTCCTGGGGTAAA | pGL3-Basic | Promoter vector  construction |
|  | R: CCGCTCGAGCATGCCGCAGGTGCACAACCTA |  |
| pGL3-SBE1-mut | F: GGCTGGCGGGGGGGTTTCGAACAACCGAGGACGTTC | pGL3-Basic | Mutation |
|  | R: TGGCCCCGCGGGCGGaaaaaGCAGCAATGTTTCCC |  |
| pGL3-SBE2-mut | F: GGGAGTGCGAGAAGCCCAATGAGCCCTCAAGGCAGCGA | pGL3-Basic | Mutation |
|  | R: GACACTGGCAGCGTCACtttttAAAGGGCCCGGGTGCG |  |  |
| pGL3-SBE3-mut | F: CCCACCGAATCTGGGCCAGAACGCTGCTCAGGCAAG | pGL3-Basic | Mutation |
|  | R: CTAGCCCCTGATCCCtttttATACTTTATCGGGCC |  |  |
| pGL3-SBE4-mut | F: CAGCCTGAGGGCGCACCTGAGAGAGGGACAGAAAT | pGL3-Basic | Mutation |
|  | R: GAAATGACAGGCGTttttGTCGGGGAGCCCAGGCTGT |  |  |
| pGL3-SBE5-mut | F: CAGGGCCACCCCAGCCCACAGCTGACTAAAGATAGCCC | pGL3-Basic | Mutation |
|  | R: GGGGACTCACCACCATTGaaaaaCACCATTGCAGCCCAA |  |
| pGL3-SBE6-mut | F: GAGGACACTTACAGTGGCTTTACCCCAGGAAGTGAAGG | pGL3-Basic | Mutation |
|  | R: ATGCACCAAGAGTAGCTGAttttCCCAGCTCAGCCT |  |  |

Mutation sites were presented with lower case letters

**Supplementary Table 6. Primers for chromatin immunoprecipitation**

| **Name** | **Primer sequence (5’ to 3’)** | **Product Length (bp)** |
| --- | --- | --- |
| Site X | P1: CAGCAAAGGGTGGCAAGGT | 145 |
|  | P2: ATGCCTGTGCCACTTTCAACT |  |
| SBE1 | P3: TCGCCCTATTCTTGTTCACC | 168 |
|  | P4: TACTGAGAACGTCCTCGGTTG |  |
| SBE2 | P5: CCGAGGACGTTCTCAGTAGA | 146 |
|  | P6: TCGGAGAGGAAGTAAACCCT |  |
| SBE3/4 | P7: ACAGCGGGCAACGGAATC | 212 |
|  | P8: CGACGTCTACGCCTGTCATTT |  |
| SBE5 | P9: GCTGGAAATGACAGGCGTAG | 212 |
|  | P10: GGACACTTACAGTGGCTTTACCC |  |
| SBE6 | P11: CTGACTAAAGATAGCCCTTCACTTC | 137 |
|  | P12: CCGCAGGTGCACAACCTA |  |
